# Exploring biomarkers of neurodegenerative risk: associations of oxysterols, sex hormones, and reproductive characteristics in older women

**DOI:** 10.1016/j.jlr.2025.100938

**Published:** 2025-11-06

**Authors:** Michelle M. Dunk, Ljerka Delac, Stephen R. Rapp, Ira Driscoll, Maria Latorre-Leal, Leslie V. Farland, Bernhard Haring, Holly R. Harris, Su Yon Jung, JoAnn E. Manson, Heather M. Ochs-Balcom, Aladdin H. Shadyab, Julie C. Weitlauf, Hong Xu, Eric Westman, Silvia Maioli

**Affiliations:** 1 Aging Research Center, Department of Neurobiology, Care Sciences and Society, Karolinska Institutet, Stockholm, Sweden; 2 Division of Neurogeriatrics, Center for Alzheimer Research, Department of Neurobiology, Care Sciences and Society, Karolinska Institutet, Stockholm, Sweden; 3 Department of Psychiatry & Behavioral Medicine, Wake Forest University School of Medicine, Winston-Salem, NC, USA; 4 Wisconsin Alzheimer's Disease Research Center, Department of Medicine, School of Medicine and Public Health, University of Wisconsin-Madison, Madison, WI, USA; 5 Department of Epidemiology and Biostatistics, Mel and Enid Zuckerman College of Public Health, University of Arizona, Tucson, AZ, USA; 6 Department of Medicine III, Saarland University, Homburg, Saarland, Germany; 7 Department of Epidemiology & Population Health, Albert Einstein College of Medicine, Bronx, NY, USA; 8 Program in Epidemiology, Division of Public Health Sciences, Fred Hutchinson Cancer Center, Seattle, WA, USA; 9 Department of Epidemiology, Fielding School of Public Health, Translational Sciences Section, Jonsson Comprehensive Cancer Center, School of Nursing, University of California, Los Angeles, CA, USA; 10 Department of Medicine, Brigham and Women’s Hospital, Harvard Medical School, Boston, MA, USA; 11 Department of Epidemiology and Environmental Health, School of Public Health and Health Professions, State University of New York at Buffalo, Buffalo, NY, USA; 12 Herbert Wertheim School of Public Health and Human Longevity Science and Division of Geriatrics, Gerontology, and Palliative Care, Department of Medicine, University of California San Diego, La Jolla, CA, USA; 13 Sierra Pacific Mental Illness, Research, Education and Clinical Center, Veterans Affairs Palo Alto Health Care System, Palo Alto, CA, USA; 14 Department of Psychiatry and Behavioral Sciences and, by courtesy, Obstetrics and Gynecology, Stanford University School of Medicine, Stanford, CA, USA; 15 Division of Clinical Geriatrics, Department of Neurobiology, Care Sciences and Society, Karolinska Institutet, Stockholm, Sweden; 16 Division of Clinical Geriatrics, Center for Alzheimer Research, Department of Neurobiology, Care Sciences and Society, Karolinska Institutet, Stockholm, Sweden

**Keywords:** lipids, hormones, cholesterol, Alzheimer’s disease, statins, 24(S)-hydroxycholesterol, CYP46A1, estrogen, apolipoprotein E, reproductive history

## Abstract

Women face a higher lifetime risk of developing neurodegenerative diseases such as Alzheimer’s disease and related dementias. The menopausal transition, characterized by a decline in estrogen levels, may affect cholesterol metabolism and neurodegenerative processes. Oxysterols, oxidized cholesterol derivatives, play a role in these pathways, with 24(S)-hydroxycholesterol (24HC) reflecting brain cholesterol turnover and 27-hydroxycholesterol (27HC) linked to systemic cholesterol metabolism. We investigated associations of plasma oxysterols with circulating sex hormones and characteristics of reproductive history in 1,974 postmenopausal women with no history of dementia from the Women’s Health Initiative, taking into account *APOE*4 status and cholesterol-lowering medication. We found that higher levels of bioavailable estradiol were associated with higher 24HC and 27HC levels, and higher estrone was associated with higher 24HC (all *P* values <0.05). Associations of estradiol with 24HC and 27HC were stronger among *APOE*4 carriers and those not using cholesterol-lowering medication, with a significant interaction between bioavailable estradiol and *APOE*4 in relation to 27HC (*p* for interaction = 0.04). Having an older age at menopause was associated with lower 24HC among those taking cholesterol medication (*p* for interaction = 0.03). Our findings suggest that 24HC and 27HC may be proxy biomarkers of neuronal health and estrogen status in postmenopausal women. The stronger associations between estradiol and oxysterols among *APOE*4 carriers and those not using cholesterol medication suggest the need to account for hormonal, genetic, and pharmacological factors when evaluating neurodegenerative risk. Longitudinal studies are warranted to further investigate oxysterols as potential early biomarkers of risk for Alzheimer’s disease and related dementias.

## Graphical Abstract

**Figure:**
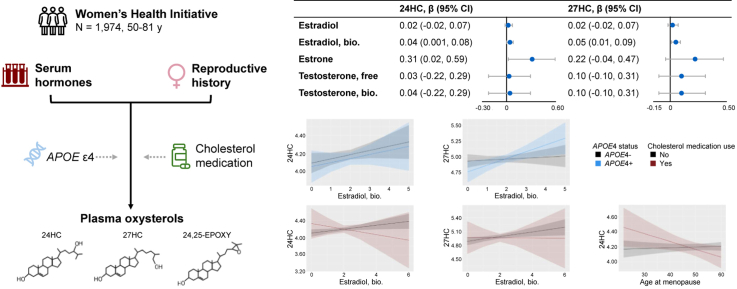

Women undergo the menopause transition during middle age, resulting in a longer postreproductive timespan than men. This menopause transition, marked by a drastic reduction in sex hormones including estrogen, is associated with cognitive decline and increased risk of neurodegeneration (1, 2). Indeed, postmenopausal women represent around two-thirds of individuals with Alzheimer’s disease (AD) and related dementias (ADRDs), a disparity that cannot be fully attributed to differences in longevity (3). Sex hormones like estrogen may differentially impact ADRD risk and pathology in women and men. For instance, earlier age at menopause, whether natural or induced, increases the risk for cognitive decline and is associated with higher levels of amyloid plaques and tau tangles, the main pathological hallmarks of AD (4). Conversely, timely intervention with estrogen-based hormone therapy (HT; at the time of menopausal symptoms) is sometimes associated with lower tau burden and decreased risk (5, 6). Moreover, hormonal changes during perimenopause, as well as other female-specific reproductive events, such as pregnancy, may interact with genetics and environmental risk factors to modulate ADRD risk and disease mechanisms (1). For instance, female carriers of the apolipoprotein E ε4 allele (*APOE*4), the most potent genetic risk factor for AD, have a higher risk of developing AD compared with male carriers and show earlier onset and faster age-related decline (7, 8, 9).

Cholesterol metabolism also appears to be a critical component of AD (10). Dysregulation of cholesterol biosynthesis, transport, and metabolism has been observed during ADRD progression (11), and hypercholesterolemia is increasingly recognized as a risk factor for AD (12, 13). Oxysterols, which are oxidized metabolites of cholesterol, play a key role in maintaining cholesterol homeostasis and can cross the blood-brain barrier (BBB) and exert biological effects through nuclear receptors, such as the liver X receptor (LXR) (14, 15). Importantly, 27-hydroxycholesterol (27HC) is a selective estrogen receptor modulator (SERM) (16), 24(S)-hydroxycholesterol (24HC) activates estrogen receptor signaling in neurons indirectly via LXR (17), and 24(S),25-epoxycholesterol (24,25-EPOXY) is an important regulator of cholesterol synthesis rate (14, 18). These oxysterols are intermediates in cholesterol metabolism and may reflect changes in brain and peripheral cholesterol turnover, providing insight into disease mechanisms. Oxysterols appear to be imbalanced in AD, signified by elevated 27HC in AD postmortem brains and decreasing 24HC levels (19). In plasma and cerebrospinal fluid (CSF), altered 24HC and 27HC were observed in AD patients (20), and increased levels of 24HC and the 24HC/27HC ratio in plasma have been associated with higher risk of cognitive impairment and dementia (21, 22).

A recent study demonstrated that overexpression of cholesterol-24(S)-hydroxylase (CYP46A1), a brain enzyme involved in 24HC and 24,25-EPOXY production, counteracts memory loss during aging and in estrogen-deprived conditions in female mice (17). Moreover, high levels of 24HC were found to correlate with lower CSF biomarkers of neurodegeneration and AD in women but not in men (17). These findings led us to hypothesize that levels of oxysterols, such as 24HC, are positively associated with estrogen levels and other female-specific reproductive factors, possibly serving as useful biomarkers for early detection of ADRD in women.

Given the limited research on relationships of oxysterols with hormonal, reproductive, and genetic factors in women, we aimed to elucidate the intricate relationships among cholesterol metabolism, sex hormones, and *APOE*. This study was conducted as a secondary analysis of the Women’s Health Initiative (WHI), a well-defined cohort of postmenopausal women. We examined cross-sectional associations of sex hormones and reproductive history with plasma oxysterols and whether these relationships varied based on *APOE*4 status and use of cholesterol-lowering medication.

## Materials and methods

### Study sample

The WHI clinical trials of HT were designed to evaluate the efficacy and safety of HT in healthy postmenopausal women for chronic disease prevention (23, 24, 25). Between 1993 and 1998, 27,347 postmenopausal women aged 50 to 81 were recruited across 40 clinical centers in the United States. In the estrogen + progestin trial, women with intact uteri were randomly assigned to active therapy of either 0.625 mg/day of conjugated equine estrogens plus 2.5 mg medroxyprogesterone acetate or placebo (23). In the estrogen-alone trial, participants with hysterectomy were randomly assigned to 0.625 mg/day of conjugated equine estrogens or placebo.

The current study comprises a subset of WHI HT participants (N = 1,974) with no history of AD and available data on oxysterols, sex hormones, and reproductive history at study recruitment, prior to HT trial initiation. Due to the limited collection of oxysterols and sex hormones, to maximize power, all participants with data on oxysterols and at least one other variable of interest (sex hormones or reproductive history) were included (see Supplemental Table S1 for available sample sizes and Supplemental Table S2 for power calculations), excluding those with a self-reported AD diagnosis at WHI enrollment (n = 3).

The WHI project is reviewed and approved by the Fred Hutchinson Cancer Research Center (Fred Hutch) Institutional Review Board (IRB) in accordance with the US Department of Health and Human Services regulations at 45 CFR 46 (approval number: IR #3467). Fred Hutch has been the single IRB of record since 2010 for all participating sites. Approval for the original study at the Clinical Coordinating Center at Fred Hutch was conducted by the Fred Hutch IRB (approval number: IR #3467) and by the original 40 clinical center site IRBs. Participants provided written informed consent to participate. Additional consent to review medical records was obtained through signed written consent. Fred Hutch has an approved FWA on file with the Office for Human Research Protections under assurance number 0001920. The trial was performed in accordance with the Declaration of Helsinki.

### Measurement of plasma oxysterols and sex hormones

Fasting plasma and serum samples at baseline were frozen and stored at −70°C (26). Concentrations of plasma oxysterols (24HC, 27HC, and 24,25-EPOXY; ng/ml) were measured via high-performance liquid chromatography-mass spectrometry at the University of Texas Southwestern Medical Center, Department of Molecular Genetics (Dallas, TX). Assay and analysis details have been published previously (27, 28). Average interassay coefficients of variability (CVs) of 24HC, 27HC, and 24,25-EPOXY were 9.5%, 9.5%, and 35.2%, respectively.

Circulating levels of serum estradiol (pg/ml) were measured using radioimmunoassay (CV, 16.5%), gas chromatography-mass spectrometry (CV, 6.5%), or liquid chromatography tandem mass spectrometry via the triple quadrupole method (CV, 11.7%) (29, 30). Estrone (pg/ml; CV, 13.6%) was measured using radioimmunoassays. Bioavailable estradiol (CV, 17.4%), free testosterone (CV, 10.3%), and bioavailable testosterone (CV, 12.0%) (pg/ml) were calculated using the methods detailed by Södergard *et al.* (31).

### Assessment of reproductive history

Characteristics of reproductive history were self-reported at WHI enrollment. The age at which participants went through menopause was determined according to an algorithm described previously (https://www.whi.org/doc/Algorithm-Age-at-Menopause.pdf). Briefly, age at menopause was defined as the age at which a participant first experienced the following: *1*) last had any menstrual bleeding, *2*) had a bilateral oophorectomy, or *3*) began using hormone replacement therapy. For those who underwent hysterectomy but not bilateral oophorectomy, age at menopause was the age at which they first began using hormone replacement therapy or first had symptoms of menopause (e.g., hot flashes, night sweats). If a participant had a hysterectomy but none of the other events (neither bilateral oophorectomy, hormone replacement therapy prior to age 60, nor menopause symptoms), then her age at menopause was defined as the age at which she had her hysterectomy. Those with an age at menopause >60 years were classified as having an age at menopause of 60.

History of oophorectomy was classified as the removal of at least one ovary and dichotomized as yes or no. Number of full-term pregnancies was defined as the number of pregnancies lasting at least 6 months, with response options of never pregnant or 0, 1, 2, 3, 4, or ≥5 full-term pregnancies. Current use of menopausal hormones (prior to WHI HT randomization) was categorized as use or nonuse of estrogen or estrogen plus progestin.

### *APOE* ε4 carrier status

*APOE* genotyping was performed on blood samples using SNPs rs429358 and rs7412. The six common *APOE* genotypes (ε2/ε2, ε2/ε3, ε2/ε4, ε3/ε3, ε3/ε4, and ε4/ε4) were determined from imputation and harmonization of genetic data across WHI genome-wide association studies: the Genomics and Randomized trials Network (GARNET), NHLBI Trans-Omics for Precision Medicine (TOPMed), WHI Memory Study+ (WHIMS+), Hip Fracture (HIPFX), SNP Health Association Resource (WHI-SHARe), Population Architecture using Genomics and Epidemiology (PAGE II), Modification of PM-mediated Arrhythmogenesis in Populations (MOPMAP), Discovery, Biology, and Risk of Inherited Variants in Breast Cancer (DRIVE Oncochip), WHI Long Life Study (LLS), and Common Variant GWAS, Genetics and Epidemiology of Colorectal Cancer Consortium (GECCO). Except where otherwise noted, participants were categorized as *APOE* ε4 (*APOE*4) carriers (ε4/ε4, ε3/ε4, or ε2/ε4) or noncarriers (ε2/ε2, ε2/ε3, or ε3/ε3).

### Ascertainment of covariates

Sociodemographic and lifestyle-related information collected via self-report at study entry included age, education (less than high school/general education development [GED], high school/GED, or more than high school/GED), smoking status (never, former, or current), and alcohol intake (weekly servings of beer, wine, or liquor according to a medium serving size [12 ounces of beer, 6 ounces of wine, and 1.5 ounces of liquor]). Medical information was gathered through self-report or standardized assessment, comprising measurement of BMI (kg/m^2^) and waist circumference (cm), current use of cholesterol-lowering medication (including bile sequestrants, fibric acid derivatives, intestinal cholesterol absorption inhibitors, hydroxymethylglutaryl-coenzyme A reductase inhibitors, nicotinic acid derivatives, and miscellaneous antihyperlipidemics), and history of hypertension, hysterectomy, cardiovascular disease, stroke, type 2 diabetes, and cancer (excluding nonmelanoma skin cancer). Global cognitive performance was assessed via the Modified Mini-Mental State (3MS) examination (32). Scores ranged from 0 to 100, with higher scores indicating better cognitive function.

### Statistical analysis

Concentrations of plasma oxysterols and serum hormones were log-transformed because of skewed distributions. Serum hormones, total cholesterol, and triglycerides were further standardized because of measurement at several different laboratories. Standardization consisted of linear regression of the log-transformed test values (performed separately for hormones, total cholesterol, and triglycerides) using the most common laboratory and assay method as reference, while also adjusting for age and ethnicity (https://www.whi.org/doc/Standardization-of-assays-from-multiple-labs-for-a-given-analyte.pdf). Test values were then adjusted according to the laboratory-specific beta coefficients from these models.

Differences in baseline characteristics according to median age at baseline (<68 vs. ≥68 years) were assessed using ANOVA for continuous variables and Chi-square tests for categorical variables. Pearson correlation coefficients were calculated to examine correlations *1*) between oxysterols and lipids and *2*) among hormones. Linear regression was performed to assess for potential differences in oxysterols and hormones according to *APOE*4 status (*APOE*4+ vs. *APOE*4-) and *APOE* genotype (ε2 [ε2/ε2, ε2/ε3], ε3 homozygotes, and ε4 [ε4/ε4, ε3/ε4], excluding ε2/ε4 carriers [n = 32] because of opposing effects of these alleles). Associations between 3MS scores and oxysterols, hormones, reproductive characteristics, and *APOE*4 status were also examined using linear regression.

Associations of oxysterols with sex hormones and reproductive history were examined using linear regression. Analysis of the number of pregnancies excluded women who had become pregnant but never reached full term (n = 56). Due to the small number of women using estrogen or estrogen + progestin, hormone use was analyzed dichotomously as use or nonuse. All models were initially adjusted for age (continuous), BMI (continuous), cholesterol-lowering medication (yes or no), oophorectomy (yes or no), hysterectomy (yes or no), and HT use (yes or no), followed by further adjustment for education (<high school/GED, high school/GED, or >high school/GED), smoking status (never, former, or current), alcohol intake (servings/week), hypertension (yes or no), cardiovascular disease (yes or no), stroke (yes or no), and type 2 diabetes (yes or no). Sensitivity analyses were performed *1*) with further adjustment for waist circumference to account for potential confounding by adiposity, which is the primary source of endogenous estrogen in postmenopausal women (33) and *2*) with further adjustment for cancer, given that oxysterols have been implicated in some cancers (34). We additionally performed interaction and stratified analyses of all models according to *APOE*4 status and use of cholesterol-lowering medication.

To account for multiple comparisons, sensitivity analyses were performed using the Benjamini-Hochberg false discovery rate (FDR) correction to *P*-values from fully-adjusted models. FDR was applied separately within each set of related models: *1*) main models examining associations between hormones and oxysterols, *2*) interaction and stratified hormone-oxysterol models by *APOE*4 status, *3*) interaction and stratified hormone-oxysterol models by cholesterol medication use, *4*) main models for reproductive variables, *5*) interaction and stratified reproductive-oxysterol models by *APOE*4 status, and *6*) interaction and stratified reproductive-oxysterol models by cholesterol medication use. This approach preserves statistical power for our already limited sample size while appropriately controlling for multiple testing within conceptually distinct hypothesis groups. Supplementary analyses of the 24HC/27HC ratio were also performed, given prior reports of increased risk of cognitive impairment and dementia among those with a higher 24HC/27HC ratio (21, 22).

Analyses were performed using R Studio, version 2023.06.1 + 524, © 2009–2023, Posit Software, PBC, with statistical significance reported at *P* < 0.05.

## Results

### Sample characteristics

Among the entire sample (N = 1,974), the mean (SD) age was 67.0 (6.9) years. Participant characteristics according to age group are presented in Table 1. Older participants (≥68 years) were more likely to have a lower BMI, hypertension, cardiovascular disease, and to take cholesterol-lowering medication, and were less likely to smoke. Older women also had lower concentrations of serum estradiol and were less likely to be taking HT. Distributions of non-log-transformed oxysterols and sex hormones are presented in Supplemental Fig. S1. 24HC levels were positively correlated with 27HC (Supplemental Table S3A). Total cholesterol levels were positively correlated with 24HC and 27HC and negatively correlated with 24,25-EPOXY. Triglyceride levels were positively correlated with 24HC, 27HC, and the 24HC/27HC ratio. All hormones were positively correlated with each other (Supplemental Table S3B).

**Table 1:** Participant characteristics

Characteristic	Pooled (N = 1,974)	Age group		
		<68 years (n = 969)	≥68 years (n = 1,005)	*P*
Age at baseline, y	67.0 ± 6.9	61.2 ± 4.6	72.5 ± 3.1	<0.001
Age at menopause, y	47.5 ± 6.9	47.3 ± 6.7	47.7 ± 7.1	0.23
Education				0.86
<High school or GED	168 (8.5)	80 (8.3)	88 (8.8)	
High school or GED	450 (22.8)	225 (23.2)	225 (22.4)	
>High school or GED	1,335 (67.6)	653 (67.4)	682 (67.9)	
BMI, kg/m^2^	28.5 ± 5.6	29.0 ± 6.1	28.0 ± 5.2	<0.001
Smoking status				<0.001
Never	993 (50.3)	431 (44.5)	562 (55.9)	
Former	727 (36.8)	372 (38.4)	355 (35.3)	
Current	213 (10.8)	150 (15.5)	63 (6.3)	
Alcohol intake, servings/wk	2.1 ± 4.8	2.1 ± 4.9	2.1 ± 4.8	0.88
Number of pregnancies				0.52
Never pregnant	142 (7.2)	63 (6.5)	79 (7.9)	
0 full-term	56 (2.8)	34 (3.5)	22 (2.2)	
1	161 (8.2)	82 (8.5)	79 (7.9)	
2	428 (21.7)	213 (22.0)	215 (21.4)	
3	433 (21.9)	216 (22.3)	217 (21.6)	
4	329 (16.7)	157 (16.2)	172 (17.1)	
≥5	419 (21.2)	200 (20.6)	219 (21.8)	
Oophorectomy	579 (29.3)	285 (29.4)	294 (29.3)	0.93
Hysterectomy	872 (44.2)	420 (43.3)	452 (45.0)	0.49
Current use of hormone therapy				<0.001
No	1832 (92.8)	871 (89.9)	961 (95.6)	
E	86 (4.4)	55 (5.7)	31 (3.1)	
E + P	55 (2.8)	43 (4.4)	12 (1.2)	
24HC, ng/ml	68.8 ± 20.2	68.4 ± 19.0	69.1 ± 21.1	0.60
27HC, ng/ml	156.0 ± 44.3	155.6 ± 43.2	156.4 ± 45.2	0.80
24,25-EPOXY, ng/ml	12.7 ± 37.7	11.2 ± 34.3	14.1 ± 40.5	0.37
Estradiol, pg/ml	12.6 ± 16.4	14.0 ± 22.8	11.4 ± 6.9	0.004
Estradiol, bioavailable, pg/ml	8.1 ± 9.0	9.2 ± 12.1	7.2 ± 4.7	<0.001
Estrone, pg/ml	43.4 ± 19.7	41.4 ± 20.9	45.0 ± 18.6	0.29
Testosterone, free, pg/ml	5.5 ± 3.0	5.7 ± 3.4	5.3 ± 2.6	0.46
Testosterone, bioavailable, pg/ml	135.6 ± 74.2	140.7 ± 83.6	130.4 ± 63.6	0.45
Hypertension	783 (39.7)	343 (35.4)	440 (43.8)	<0.001
Total cholesterol, mg/dl	232.4 ± 39.2	230.9 ± 38.4	234.0 ± 39.9	0.08
Triglycerides, mg/dl	147.5 ± 83.5	145.6 ± 84.1	149.4 ± 82.9	0.31
Cardiovascular disease	356 (18.0)	146 (15.1)	210 (20.9)	0.002
Type 2 diabetes mellitus	200 (10.1)	102 (10.5)	98 (9.8)	0.63
Stroke	38 (1.9)	19 (2.0)	19 (1.9)	1.00
*APOE* ε4 carrier	429 (21.7)	217 (22.4)	212 (21.1)	0.11
Cholesterol-lowering medication	197 (10.0)	73 (7.5)	124 (12.3)	<0.001
Baseline 3MS score	94.6 ± 4.7	94.7 ± 5.3	94.6 ± 4.6	0.73

Values are M ± SD or N (%). Differences in baseline characteristics by age were assessed using ANOVA for continuous variables and Chi-square tests for categorical variables.

*Missing data*: 24HC = 1,120; 24–25 epoxycholesterol = 1,446; 27HC = 1,120; age at menopause = 266; alcohol intake = 14; *APOE* ε4 status = 353; baseline 3MS score = 1,144; BMI = 6; cardiovascular disease = 243; current use of hormone therapy = 1; education = 21; estradiol = 683; estradiol, bioavailable = 692; estrone = 1,836; hypertension = 17; number of pregnancies = 6; oophorectomy = 37; smoking status = 41; testosterone, bioavailable = 1,855; testosterone, free = 1,855; type 2 diabetes mellitus = 1.

E, estrogen; E + P, estrogen plus progestin.

A total of 1,621 women (82%) were genotyped for *APOE*, of whom 429 (21.7%) were *APOE*4 carriers and 1,192 (60.4%) were noncarriers. Oxysterols did not differ significantly between *APOE*4 carriers and noncarriers (Supplemental Table S4), although analysis of *APOE* genotypes revealed higher 24,25-EPOXY levels among ε2 compared with ε3 carriers (Supplemental Table S5). *APOE*4 carriers had significantly higher levels of estradiol and bioavailable estradiol compared with noncarriers (Supplemental Table S4). In analysis of *APOE* genotypes, ε4 carriers had higher estradiol levels, whereas ε2 carriers had higher 24,25-EPOXY (Supplemental Table S5). *APOE*4 carriers had significantly lower global cognitive function as measured by 3MS scores. Higher 27HC was associated with lower cognitive function after basic adjustment, although this was attenuated following multivariable adjustment (Supplemental Table S6).

### Relationships between sex hormones and oxysterols

Higher levels of bioavailable estradiol (β [95% CI] = 0.04 [0.001, 0.08], *P* = 0.046) and estrone (0.31 [0.02, 0.59], *P* = 0.04) were significantly associated with higher 24HC (Table 2). Higher 27HC levels were also observed in relation to higher bioavailable estradiol (0.05 [0.01, 0.09], *P* = 0.02) though not estrone (0.22 [-0.04, 0.47], *P* = 0.09). No significant associations were detected between 24HC or 27HC and estradiol, free testosterone, or bioavailable testosterone (*ps* ≥ 0.26), and 24,25-EPOXY was not associated with any sex hormones (*ps* ≥ 0.45). These results remained similar after adjustment for waist circumference, although the association between bioavailable estradiol and 24HC was attenuated (*P* = 0.06) (Supplemental Table S7). Associations between hormones and oxysterols also remained robust after further adjustment for cancer, along with an additional positive association between estrone and 27HC (0.27 [0.01, 0.52], *P* = 0.04) (Supplemental Table S8).

**Table 2:** Associations between sex hormones and oxysterols

Hormone	24HC		27HC		24,25-EPOXY	
	β (95% CI)	*P*	β (95% CI)	*P*	β (95% CI)	*P*
Estradiol	0.02 (−0.02, 0.07)	0.27	0.02 (−0.02, 0.07)	0.26	−2.28 (−9.67, 5.10)	0.54
Estradiol ∗ *APOE*4	−0.001 (−0.10, 0.10)	0.98	0.09 (−0.003, 0.19)	0.06	15.31 (−9.42, 40.03)	0.22
Estradiol ∗ cholesterol med.	−0.14 (−0.32, 0.03)	0.11	−0.09 (−0.26, 0.08)	0.30	−21.06 (−46.46, 4.34)	0.10
Estradiol (B)	**0.04 (0.001, 0.08)**	**0.046**	**0.05 (0.01, 0.09)**	**0.02**	−2.31 (−9.45, 4.82)	0.52
Estradiol (B) ∗ *APOE*4	−0.002 (−0.09, 0.09)	0.97	**0.09 (0.004, 0.18)**	**0.04**	12.76 (−9.35, 34.87)	0.26
Estradiol (B) ∗ cholesterol med.	−0.11 (−0.28, 0.06)	0.20	−0.05 (−0.22, 0.11)	0.53	−19.21 (−43.88, 5.45)	0.13
Estrone	**0.31 (0.02, 0.59)**	**0.04**	0.22 (−0.04, 0.47)	0.09	20.60 (−44.74, 85.94)	0.52
Estrone ∗ *APOE*4	−0.06 (−0.77, 0.65)	0.87	0.02 (−0.60, 0.64)	0.94	63.75 (−137.17, 264.66)	0.51
Estrone ∗ cholesterol med.	0.13 (−1.06, 1.32)	0.83	0.19 (−0.86, 1.24)	0.72	5.38 (−231.09, 241.85)	0.96
Testosterone (F)	0.03 (−0.22, 0.29)	0.79	0.10 (−0.10, 0.31)	0.32	25.69 (−44.38, 95.76)	0.45
Testosterone (F) ∗ *APOE*4	0.15 (−0.86, 1.16)	0.77	−0.03 (−0.89, 0.84)	0.95	−71.93 (−623.94, 480.09)	0.78
Testosterone (F) ∗ cholesterol med.	−0.47 (−1.53, 0.60)	0.38	−0.73 (−1.55, 0.08)	0.08	−16.90 (−253.90, 220.11)	0.88
Testosterone (B)	0.04 (−0.22, 0.29)	0.78	0.10 (−0.10, 0.31)	0.31	25.67 (−44.40, 95.73)	0.45
Testosterone (B) ∗ *APOE*4	0.15 (−0.87, 1.16)	0.77	−0.02 (−0.89, 0.84)	0.95	−72.14 (−624.76, 480.48)	0.77
Testosterone (B) ∗ cholesterol med.	−0.47 (−1.54, 0.60)	0.38	−0.74 (−1.55, 0.08)	0.08	−16.87 (−254.08, 220.35)	0.88

Models were adjusted for age, BMI, cholesterol-lowering medication (except in cholesterol medication interaction models), oophorectomy, hysterectomy, hormone use, education, smoking status, alcohol intake, hypertension, cardiovascular disease, stroke, and type 2 diabetes. Bold values indicate statistical significance.

B, bioavailable; cholesterol med., cholesterol-lowering medication; F, free.

**Fig. 1:**
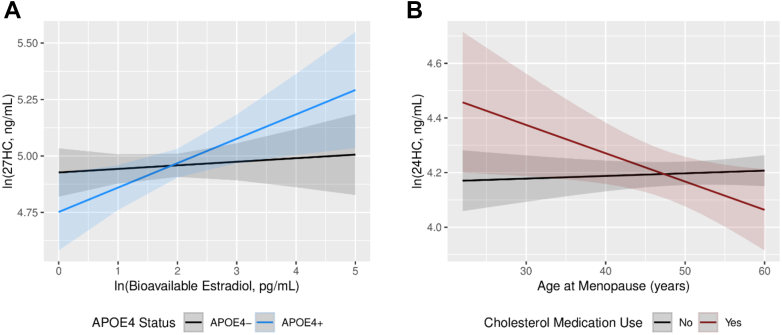
Associations between (A) bioavailable estradiol and 27HC according to *APOE*4 status and (B) age at menopause and 24HC according to cholesterol medication use. Models were adjusted for age, BMI, cholesterol-lowering medication (except in plot B), oophorectomy, hysterectomy, hormone use, education, smoking status, alcohol intake, hypertension, cardiovascular disease, stroke, and type 2 diabetes.

**Fig. 2:**
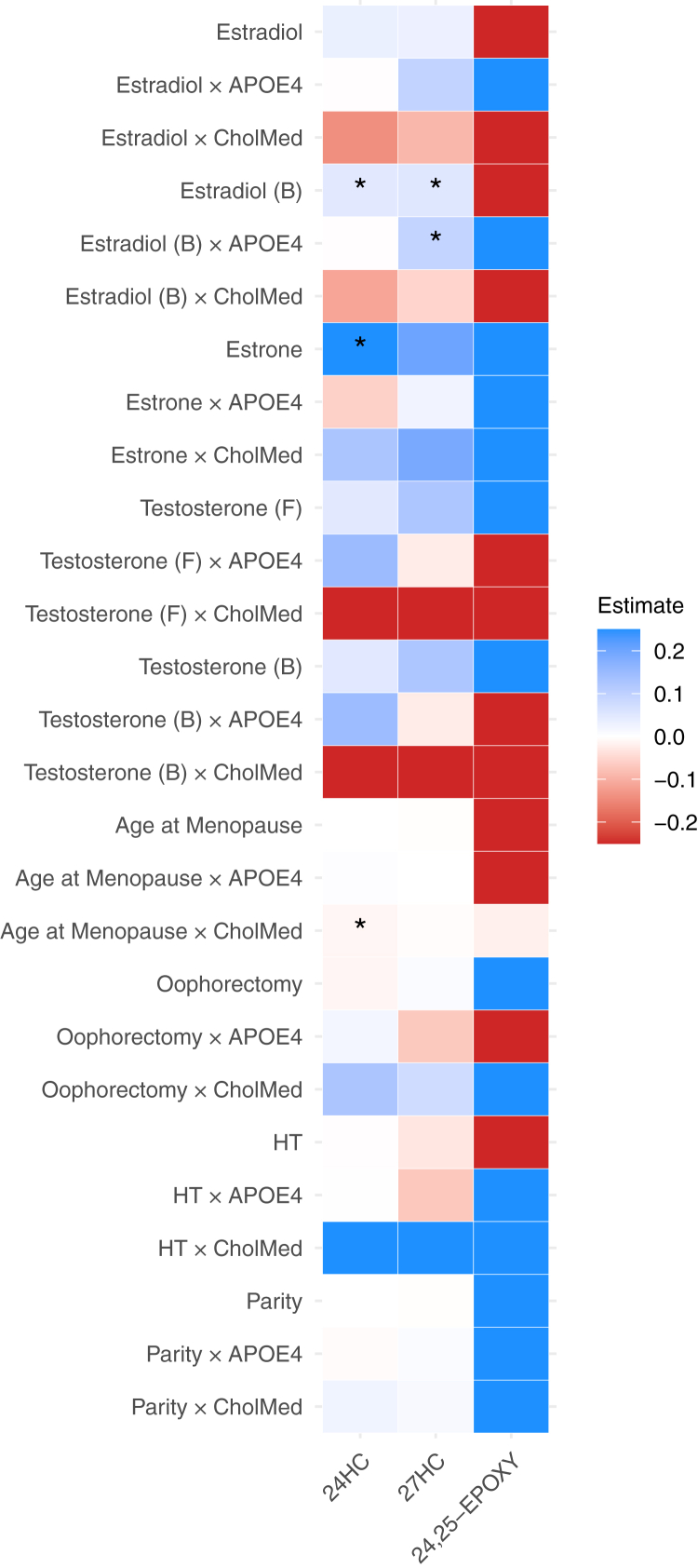
Heatmap of associations between hormones, reproductive characteristics, and oxysterols and their interactions with *APOE* and cholesterol medication use. Models were adjusted for age, BMI, cholesterol-lowering medication (except in cholesterol medication interaction models), oophorectomy, hysterectomy, hormone use, education, smoking status, alcohol intake, hypertension, cardiovascular disease, stroke, and type 2 diabetes. B, bioavailable; CholMed, cholesterol-lowering medication; F, free; HT, hormone use.

There was a significant interaction between *APOE*4 status and bioavailable estradiol in relation to 27HC (0.09 [0.004, 0.18], *p* for interaction = 0.04), such that positive associations between bioavailable estradiol and 27HC were observed only among *APOE*4 carriers (0.16 [0.05, 0.27], *P* = 0.004) (Table 2, Fig. 1A, Fig. 2, and Supplemental Table S9). A marginally significant interaction between *APOE*4 status and estradiol was also observed (0.09 [−0.003, 0.19], *p* for interaction = 0.06), with a positive association between estradiol and 27HC only for *APOE*4 carriers (0.13 [0.02, 0.24], *P* = 0.02). These interactions were attenuated after adjusting for waist circumference (Supplemental Table S7) but remained significant following adjustment for cancer (Supplemental Table S8). In stratified analysis, a positive association between bioavailable estradiol and 24HC was also detected among *APOE*4 carriers (0.11 [0.01, 0.22], *P* = 0.04). Further differences were observed among women not using cholesterol medication, including positive associations of bioavailable estradiol with both 24HC (0.05 [0.01, 0.09], *P* = 0.02) and 27HC (0.06 [0.01, 0.10], *P* = 0.008) (Supplemental Table S9). Among those taking cholesterol medication, higher estradiol (−24.01 [−47.47, −0.56], *P* = 0.045) and bioavailable estradiol (−27.54 [−52.05, −3.03], *P* = 0.03) were associated with lower levels of 24,25-EPOXY.

Significant associations between hormones and oxysterols were attenuated after FDR correction for multiple testing (Supplemental Table S10).

In supplementary analysis of the 24HC/27HC ratio, significant interactions were detected between *APOE*4 status and estradiol and bioavailable estradiol, with *APOE*4 carriers exhibiting negative associations (estradiol: −0.02 [−0.03, −0.0002], *p* for interaction = 0.047; bioavailable estradiol: −0.02 [−0.03, −0.001], *p* for interaction = 0.03) (Supplemental Table S11).

### Relationships between reproductive history and oxysterols

There were no differences in 24HC, 27HC, or 24,25-EPOXY based on age at menopause, history of oophorectomy, number of pregnancies, or HT use (*p*s ≥ 0.13; Table 3). Results were similar after further adjustment for waist circumference (Supplemental Table S12) and cancer (Supplemental Table S13).

**Table 3:** Associations between reproductive history and oxysterols

Reproductive history	24HC		27HC		24,25-EPOXY	
	β (95% CI)	*P*	β (95% CI)	*P*	β (95% CI)	*P*
Age at menopause	−0.0003 (−0.004, 0.003)	0.86	−0.002 (−0.01, 0.001)	0.24	−0.33 (−0.95, 0.30)	0.30
Age at menopause ∗ *APOE*4	0.004 (−0.005, 0.01)	0.41	−0.001 (−0.01, 0.01)	0.89	−1.73 (−3.65, 0.19)	0.08
Age at menopause ∗ cholesterol med.	**−0.01 (-0.02, −0.001)**	**0.03**	−0.004 (−0.01, 0.01)	0.42	−0.02 (−1.58, 1.54)	0.98
Oophorectomy	−0.01 (−0.07, 0.04)	0.64	0.01 (−0.04, 0.07)	0.69	7.85 (−2.42, 18.11)	0.13
Oophorectomy ∗ *APOE*4	0.02 (−0.10, 0.14)	0.74	−0.07 (−0.18, 0.04)	0.24	−3.90 (−31.49, 23.68)	0.78
Oophorectomy ∗ cholesterol med.	0.13 (−0.04, 0.29)	0.13	0.08 (−0.08, 0.23)	0.35	3.85 (−23.81, 31.51)	0.79
Current hormone use	0.003 (−0.08, 0.08)	0.95	−0.03 (−0.10, 0.05)	0.47	−11.35 (−26.17, 3.47)	0.13
Current hormone use ∗ *APOE*4	0.002 (−0.20, 0.21)	0.99	−0.07 (−0.27, 0.13)	0.48	13.83 (−37.29, 64.94)	0.59
Current hormone use ∗ cholesterol med.	0.52 (−0.05, 1.08)	0.07	0.36 (−0.19, 0.91)	0.20	13.82 (−65.85, 93.50)	0.73
Number of pregnancies	0.002 (−0.01, 0.02)	0.75	−0.002 (−0.02, 0.01)	0.78	0.69 (−1.88, 3.25)	0.60
Number of pregnancies ∗ *APOE*4	−0.004 (−0.04, 0.03)	0.82	0.01 (−0.03, 0.04)	0.66	3.23 (−5.43, 11.88)	0.46
Number of pregnancies ∗ cholesterol med.	0.02 (−0.03, 0.08)	0.37	0.01 (−0.04, 0.06)	0.60	2.24 (−6.50, 10.98)	0.61

Models were adjusted for age, BMI, cholesterol-lowering medication (except in cholesterol medication interaction models), oophorectomy (except in models examining oophorectomy), hysterectomy, hormone use (except in models examining hormone use), education, smoking status, alcohol intake, hypertension, cardiovascular disease, stroke, and type 2 diabetes. Bold values indicate statistical significance.

Cholesterol med., cholesterol-lowering medication.

No interactions were detected between reproductive characteristics and *APOE*4 status in relation to oxysterols (*p*s for interaction ≥ 0.08). There was a significant interaction between age at menopause and cholesterol medication in relation to 24HC, where an older age at menopause was associated with lower 24HC only among those taking cholesterol medication (−0.01 [−0.02, −0.001], *p* for interaction = 0.03) (Table 3, Fig. 1B, and Supplemental Table S14). This interaction remained robust after adjusting for waist circumference (Supplemental Table S12) and cancer (Supplemental Table S13).

Associations between reproductive history and oxysterols were attenuated after adjustment for multiple comparisons (Supplemental Table S15).

Supplementary analysis of the 24HC/27HC ratio revealed no significant associations with reproductive characteristics (Supplemental Table S16). No interactions were detected according to *APOE*4 status or use of cholesterol-lowering medication, aside from a marginally significant interaction between age at menopause and cholesterol medication (−0.002 [−0.003, 0.0001], *P* = 0.06).

## Discussion

In the present study, we investigated cross-sectional associations between sex hormones, reproductive history, and oxysterols in a sample of postmenopausal women, and whether these associations varied based on *APOE*4 status or use of cholesterol medication. Oxysterols, particularly 24HC and 27HC, have been proposed as biomarkers of neurodegeneration, especially in the context of AD (20), and our aim was to assess whether they correlated with female-unique factors known to affect the risk of dementia (35).

We found that bioavailable estradiol (free and albumin-bound estradiol) was positively associated with 24HC and 27HC levels. Higher estrone levels were also associated with higher circulating 24HC but not 27HC. Elevated levels of 24HC have been reported to sustain brain estrogen signaling via LXR activation and counteract the negative effects of ovariectomy on memory in mice (17). Additionally, high CSF 24HC levels have been observed to correlate with a lower burden of AD biomarkers in women only (17). High levels of BBB-permeable 24HC in plasma may reflect elevated levels in the brain, potentially sustaining estrogen signaling in postmenopausal women. We speculate that this sustained estrogen signaling may, in turn, be reflected by higher levels of bioavailable estradiol in serum, suggesting that 24HC levels in serum could serve as a potential biomarker for estrogen-related health status in women. Future longitudinal studies exploring the relationships between plasma and CSF 24HC and estrogen levels, and how 24HC levels vary before and after menopause, would help clarify and interpret these findings.

On the other hand, 27HC is an SERM that can diminish estrogen's protective effects on bone and endothelial cells (16, 36). Thus, one may have expected a negative correlation. A case-cohort study in the WHI demonstrated a relationship between 27HC and estradiol in the context of bone fracture risk, where higher 27HC relative to bioavailable estradiol was associated with increased fracture risk (27). The positive association between 27HC serum levels and bioavailable estradiol in our study could potentially be explained by a compensatory mechanism in which elevated 27HC, because of its SERM activity, reduces estrogen's protective effects, prompting the body to maintain or increase bioavailable estradiol levels. A large cross-sectional study of premenopausal and postmenopausal women, however, found higher 27HC levels in the postmenopausal subset (37), where estrogen levels are lower. This suggests that the relationship between estrogen levels and 27HC is not connected in a causative pathway but may be influenced by the metabolic and lipid changes known to occur during and after menopause (38). Further research is needed to investigate these mechanisms and elucidate the interplay between 27HC and estrogen signaling in a longitudinal analysis.

Importantly, most associations between 24HC and 27HC and hormones remained significant after further adjusting for waist circumference, a proxy for adiposity, which is known to affect estrogen production (33). These associations also appeared to be independent of cardiovascular disease and cancer, two conditions known to interact with oxysterol levels, especially with 27HC. Further investigation using larger samples is warranted to clarify whether these diseases might alter these relationships, including in the context of ADRD risk.

Women between the ages of 60 and 70 who are *APOE*4 carriers have a higher risk of developing AD compared with noncarriers (39). *APOE*4 carriers tend to have less favorable lipid profiles, including in the WHI (40, 41, 42). *APOE*4 may modify associations between hormones and oxysterols, as shown by the significant interaction of *APOE*4 with estradiol and our observation that estradiol was positively associated with 27HC only in women with *APOE*4. Although the interaction was not significant for 24HC, stratified analysis similarly showed that estradiol was associated with 24HC exclusively among *APOE*4 carriers. *APOE*4 is thought to interact with sex hormone changes during menopause, which may in turn increase the risk for neurodegeneration (43). *APOE*4 carriers in this cohort had higher estradiol levels compared with noncarriers, and both *APOE*4 and higher 27HC levels were associated with poorer global cognition. While it is unclear why *APOE*4 carriers had higher levels of circulating estradiol, higher estradiol levels in *APOE*4 carriers have previously been associated with accelerated brain aging (44), and 27HC has also been linked to poorer cognition in prior studies (45). Research on the impact of HT on ADRD risk is mixed, with some reports of potential benefits, harms, or no associations (46, 47). Some studies reporting benefits suggest they may be exclusive to *APOE*4 carriers (48), whereas others report protection only among noncarriers (49), signaling the need for more studies. While further research is needed to clarify underlying mechanisms, our results may reflect a critical interplay between oxysterols, hormones, and *APOE*4 in postmenopausal women. These findings are consistent with some previous reports in the context of ADRD in animal models and smaller clinical cohorts (17, 43, 44, 45), underscoring a potentially modifiable pathway of ADRD risk.

In a subset of women using hypolipidemic treatment, an older age at menopause was associated with significantly lower 24HC levels in comparison to the medication-free group. Moreover, women not using hypolipidemic medication exhibited stronger positive associations of bioavailable estradiol with 24HC and 27HC. Treatment with statins has been reported to reduce 24HC levels in the plasma of AD patients, although these studies were performed on small sample sizes (50, 51, 52, 53). One study further found that this effect was irrespective of *APOE* genotype (52). Interestingly, Vega *et al.* demonstrated that female AD patients have higher baseline 24HC levels in comparison to men (53). Reduction of cholesterol synthesis in the CNS because of hypolipidemic treatment could consequently lower 24HC levels that are mirrored in peripheral readouts. However, short-term simvastatin treatment did not affect 24HC levels in the CSF (52). We did not perform additional stratification per antilipemic drug (and their BBB permeability) because of limited power. Longitudinal studies monitoring midlife hypercholesterolemia, duration, and type of hypolipidemic treatments would clarify whether 24HC can be used as a reliable marker in this cohort. Additional factors such as number of pregnancies and cholesterol medication can modify levels of oxysterols and should be taken into consideration when studying oxysterols as prospective biomarkers for dementia risk in women.

Several study limitations should be acknowledged. First, some analyses—especially those examining interactions and stratification by *APOE*—may be underpowered because of limited data collection. Several stratified models were also not estimable because of very small sample sizes, leading to unstable or nonidentifiable estimates. The observed associations between hormones, reproductive history, and oxysterols were no longer significant after FDR correction, which may be related to the limited power of this exploratory study. Further investigations in larger samples are therefore needed to corroborate our findings and verify whether associations may differ according to *APOE*4 status. Similarly, further distinction between *APOE* ε2, ε3, and ε4 alleles in future larger-scale studies is needed. Second, the lack of oxysterol and hormone measurement over follow-up in WHI prevented us from assessing changes in associations over time. Third, the high CV for 24,25-EPOXY indicates low precision, so these results should be interpreted with caution. Fourth, oxysterols are involved in and influenced by various cellular mechanisms and diseases, including oxidative stress, inflammation, cancer, and cardiovascular disease (34). *APOE* has also been implicated in cardiovascular disease, type 2 diabetes, and other neurological diseases. While we adjusted for these diseases in analysis to control for potential confounding, these conditions could impact pathogenic mechanisms, adding further complexity to the interpretation of our results. Fifth, the WHI consists only of postmenopausal women, so future investigations are needed to clarify how relationships between oxysterols, hormones, and reproductive characteristics may vary across different stages of menopause. Despite these shortcomings, to our knowledge, this study is the first to examine associations between sex hormones, reproductive characteristics, and oxysterols in a well-characterized sample of postmenopausal women. The collection of plasma oxysterols is not typically available in many cohort studies, and our sample is considerably larger than previously published studies of oxysterols in human subjects.

In summary, we observed statistically significant positive associations between serum estradiol and plasma 24HC and 27HC in this cohort of postmenopausal women. These associations were most prominent in women at higher risk, namely *APOE*4 carriers and those not using cholesterol medication. Our findings build upon the growing evidence suggesting that oxysterols play a major role in maintaining cholesterol homeostasis and may be critically involved in processes underlying ADRD. These results reinforce the possibility that 24HC and 27HC could serve as proxy biomarkers of estrogen status and ADRD risk in older women. Additionally, the limited associations between oxysterols, testosterone, and other reproductive characteristics suggest that estradiol may represent the most relevant pathway for future mechanistic studies in the context of oxysterols and ADRD. While larger-scale and longitudinal studies of oxysterols in humans are needed, our findings underscore the importance of considering genetic, vascular, and pharmacological factors when evaluating neurodegenerative risk.

## Data availability

All data supporting the findings of this study are included in the article and supplemental data. Data used for the present study are the property of the National Institutes of Health. Copies of the deidentified data used in our study can be made available upon request to, and pending approval by, the WHI Publications and Presentations Committee (email: p&p@whi.org).

### Short list of WHI investigators

*Program Office*: (National Heart, Lung, and Blood Institute, Bethesda, MD) Jacques Rossouw, Jared Reis, and Candice Price.

*Clinical Coordinating Center*: (Fred Hutchinson Cancer Center, Seattle, WA) Garnet Anderson, Ross Prentice, Andrea LaCroix, and Charles Kooperberg.

*Steering Committee and Academic Centers*: (University of Alabama at Birmingham) Gretchen Wells; (Albert Einstein College of Medicine) Yasmin Mossavar-Rahmani; (University at Buffalo) Amy Millen; (University at Buffalo) Jean Wactawski-Wende; (Fred Hutchinson Cancer Center) Marian Neuhouser; (Fred Hutchinson Cancer Center) Holly Harris; (University of Massachusetts) Brian Silver; (University of North Carolina) Nora Franceschini; (Stanford Prevention Research Center) Marcia L. Stefanick; (The Ohio State University) Electra Paskett; and (Wake Forest University) Mara Vitolins.

For a list of all the investigators who have contributed to WHI science, please visit: https://s3-us-west-2.amazonaws.com/www-whi-org/wp-content/uploads/WHI-Investigator-Long-List.pdf.

## Supplemental data

This article contains supplemental data.

## Conflict of interest

The authors declare that they have no conflicts of interest with the contents of this article.
