# Supplementary material for: Exploring biomarkers of neurodegenerative risk: associations of oxysterols, sex hormones, and reproductive characteristics in older women
Source: J Lipid Res. 2025 Nov 6;66(12):100938. doi: 10.1016/j.jlr.2025.100938 (PMC12863040; doi:10.1016/j.jlr.2025.100938)
Supplement: Supplemental Figure and Tables [file mmc1.pdf]

Supplemental Material

Supplemental Table S1. Sample sizes for analysis.

Supplemental Table S2. Power calculations**.**

Supplemental Figure S1. Distributions of oxysterols and sex hormones in the study sample.

Supplemental Table S3. Pearson correlation coefficients of oxysterols, lipids, and sex hormones.

Supplemental Table S4. Concentrations of oxysterols and sex hormones according to *APOE*4 status.

Supplemental Table S5. Concentrations of oxysterols and sex hormones according to *APOE* genotype.

Supplemental Table S6. Associations between cognitive status and oxysterols, hormones, reproductive characteristics, and *APOE*.

Supplemental Table S7. Associations between sex hormones and oxysterols after further adjustment for waist circumference.

Supplemental Table S8. Associations between sex hormones and oxysterols after further adjustment for cancer.

Supplemental Table S9. Associations between sex hormones and oxysterols according to *APOE*4 status and cholesterol medication use.

Supplemental Table S10. False Discovery Rate-adjusted *p*-values for associations between sex hormones and oxysterols.

Supplemental Table S11. Associations between sex hormones and 24HC/27HC ratio.

Supplemental Table S12. Associations between reproductive history and oxysterols after further adjustment for waist circumference.

Supplemental Table S13. Associations between reproductive history and oxysterols after further adjustment for cancer.

Supplemental Table S14. Associations between reproductive history and oxysterols according to *APOE*4 status and cholesterol medication use.

Supplemental Table S15. False Discovery Rate-adjusted *p*-values for associations between reproductive history and oxysterols.

Supplemental Table S16. Associations between reproductive history and 24HC/27HC ratio.

Supplemental Table S1. Sample sizes for analysis.

Variable	24HC	27HC	24,25-EPOXY	24HC/27HC
**Hormone**				
Estradiol	797	797	495	797
Estradiol (B)	796	796	494	796
Estrone	78	78	52	78
Testosterone (F)	57	57	40	57
Testosterone (B)	57	57	40	57
**Reproductive characteristic**				
Age at menopause	753	753	467	753
Oophorectomy	842	842	522	842
No	577	577	363	577
Yes	265	265	159	265
Hormone use	853	853	527	853
No	786	786	489	786
Yes	67	67	38	67
E	45	45	24	45
E+P	22	22	14	22
Number of pregnancies	823	823	514	823
Never pregnant	68	68	37	68
1	69	69	38	69
2	182	182	116	182
3	192	192	123	192
4	142	142	96	142
≥5	170	170	104	170

Abbreviations: 24,25-EPOXY, 24(S)-25-epoxycholesterol; 24HC, 24(S)-hydroxycholesterol; 27HC, 27-hydroxycholesterol; B, bioavailable; E, estrogen; F, free; P, progestin.

Supplemental Table S2. Power calculations.

Independent variable	Model adjustment	Power	Effect size	Sample size
**Sex hormone**	Basic adjustment	0.80	0.15	98
			0.35	46
		0.90	0.15	123
			0.35	57
	Multivariable adjustment	0.80	0.15	139
			0.35	68
		0.90	0.15	171
			0.35	81
**Reproductive history**				
	Basic adjustment	0.80	0.15	98
			0.35	46
		0.90	0.15	123
			0.35	57
	Multivariable adjustment	0.80	0.15	139
			0.35	68
		0.90	0.15	171
			0.35	81

Basic adjustment included age, body mass index, cholesterol-lowering medication, oophorectomy, hysterectomy, and hormone use. Multivariable adjustment further included education, smoking status, alcohol intake, hypertension, cardiovascular disease, stroke, and type 2 diabetes.

Supplemental Figure S1. Distribution of oxysterols and sex hormones in the study sample.

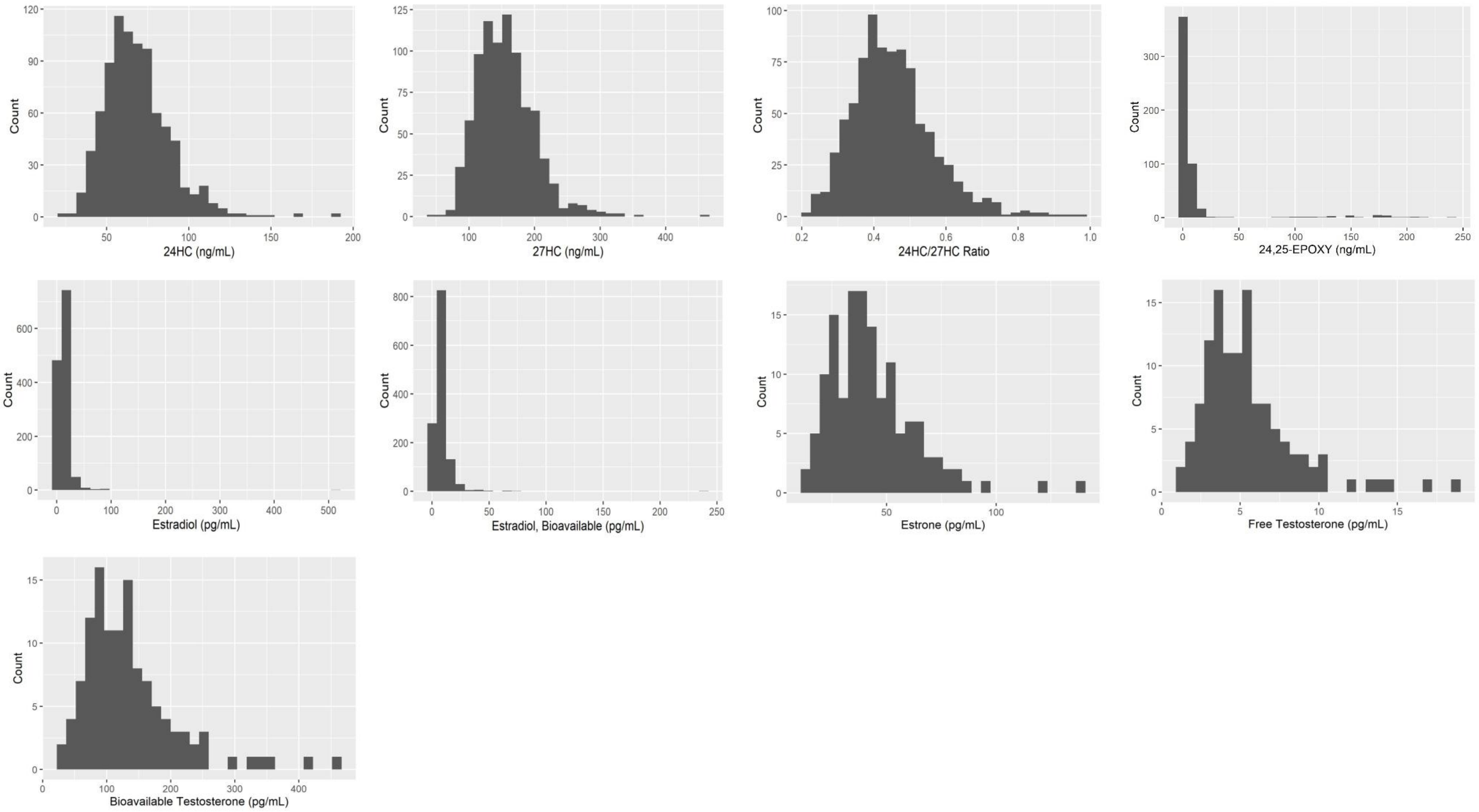

Abbreviations: 24,25-EPOXY, 24(S),25-epoxycholesterol; 24HC, 24(S)-hydroxycholesterol; 27HC, 27-hydroxycholesterol.

Supplemental Table S3. Pearson correlation coefficients of oxysterols, lipids, and sex hormones.

A.

	24HC	27HC	24HC/27HC	24,25-EPOXY	Total cholesterol	Triglycerides
**24HC**	1.00	**0.61*** **	**0.61*** **	0.05	**0.52*** **	**0.31*** **
**27HC**	**0.61*** **	1.00	**-0.26*** **	0.03	**0.57*** **	**0.29*** **
**24HC/27HC**	**0.61*** **	**-0.26*** **	1.00	0.02	0.06	**0.08* **
**24,25-EPOXY**	0.05	0.03	0.02	1.00	**-0.09* **	0.08
**Total cholesterol**	**0.52*** **	**0.57*** **	0.06	**-0.09* **	1.00	**0.31*** **
**Triglycerides**	**0.31*** **	**0.29*** **	**0.08* **	0.08	**0.31*** **	1.00

Abbreviations: 24,25-EPOXY, 24(S),25-epoxycholesterol; 24HC, 24(S)-hydroxycholesterol; 27HC, 27-hydroxycholesterol.
*$p < 0.05$; **$p < 0.01$; ***$p < 0.001$.

B.

	Estradiol	Estradiol (B)	Estrone	Testosterone (F)	Testosterone (B)
**Estradiol**	1.00	**0.95*** **	**0.68*** **	**0.47*** **	**0.47*** **
**Estradiol (B)**	**0.95*** **	1.00	**0.65*** **	**0.43*** **	**0.43*** **
**Estrone**	**0.68*** **	**0.65*** **	1.00	**0.56*** **	**0.56*** **
**Testosterone (F)**	**0.47*** **	**0.43*** **	**0.56*** **	1.00	**1.00*** **
**Testosterone (B)**	**0.47*** **	**0.43*** **	**0.56*** **	**1.00*** **	1.00

Abbreviations: B, bioavailable; F, free.
*$p < 0.05$; **$p < 0.01$; ***$p < 0.001$.

Supplemental Table S4. Concentrations of oxysterols and sex hormones according to *APOE*4 status.

Variable	Basic adjustment LSM (SE)	β (95% CI)	*p*-value	Multivariable adjustment LSM (SE)	β (95% CI)	*p*-value
**Oxysterol**						
24HC						
*APOE*4-	4.19 (0.03)	Reference		4.20 (0.11)	Reference	
*APOE*4+	4.19 (0.03)	0.004 (-0.05, 0.05)	0.89	4.16 (0.11)	-0.04 (-0.09, 0.02)	0.19
27HC						
*APOE*4-	4.98 (0.03)	Reference		5.06 (0.11)	Reference	
*APOE*4+	5.01 (0.03)	0.03 (-0.02, 0.08)	0.21	5.06 (0.11)	0.01 (-0.05, 0.06)	0.83
24,25-EPOXY						
*APOE*4-	16.06 (6.34)	Reference		7.77 (19.70)	Reference	
*APOE*4+	6.25 (7.46)	-9.82 (-20.84, 1.21)	0.08	-0.48 (19.50)	-8.25 (-20.97, 4.48)	0.20
24HC/27HC						
*APOE*4-	0.84 (0.005)	Reference		0.83 (0.02)	Reference	
*APOE*4+	0.84 (0.01)	-0.004 (-0.01, 0.004)	0.30	0.82 (0.02)	-0.01 (-0.02, 0.001)	0.07
**Hormone**						
Estradiol						
*APOE*4-	2.35 (0.04)	Reference		2.35 (0.09)	Reference	
*APOE*4+	2.42 (0.04)	**0.07 (0.002, 0.14)**	**0.04**	2.43 (0.09)	**0.09 (0.01, 0.16)**	**0.03**
Estradiol (B)						
*APOE*4-	1.90 (0.04)	Reference		1.94 (0.09)	Reference	
*APOE*4+	1.96 (0.05)	0.06 (-0.01, 0.13)	0.09	2.02 (0.09)	**0.09 (0.01, 0.17)**	**0.03**
Estrone						
*APOE*4-	3.72 (0.11)	Reference		3.59 (0.27)	Reference	
*APOE*4+	3.70 (0.13)	-0.02 (-0.19, 0.15)	0.85	3.55 (0.29)	-0.04 (-0.24, 0.17)	0.72
Testosterone (F)						
*APOE*4-	1.75 (0.13)	Reference		1.27 (0.32)	Reference	
*APOE*4+	1.76 (0.16)	0.003 (-0.22, 0.23)	0.98	1.32 (0.35)	0.05 (-0.21, 0.31)	0.70
Testosterone (B)						
*APOE*4-	4.95 (0.13)	Reference		4.47 (0.32)	Reference	
*APOE*4+	4.96 (0.16)	0.003 (-0.23, 0.23)	0.98	4.52 (0.35)	0.05 (-0.21, 0.31)	0.70

Basic adjustment included age, body mass index, cholesterol-lowering medication, oophorectomy, hysterectomy, and hormone use. Multivariable adjustment further included education, smoking status, alcohol intake, hypertension, cardiovascular disease, stroke, and type 2 diabetes. *Abbreviations:* 24,25-EPOXY, 24(S),25-epoxycholesterol; 24HC, 24(S)-hydroxycholesterol; 27HC, 27-hydroxycholesterol; *APOE*4+, Apolipoprotein E ε4 carrier; *APOE*4-, Apolipoprotein E ε4 non-carrier; B, bioavailable; CI, confidence interval; F, free; LSM, least-squares mean; SE, standard error.

Supplemental Table S5. Concentrations of oxysterols and sex hormones according to *APOE* genotype.

Variable	Basic adjustment LSM (SE)	β (95% CI)	*p*-value	Multivariable adjustment LSM (SE)	β (95% CI)	*p*-value
**Oxysterol**						
24HC						
*APOE*2	4.14 (0.04)	-0.05 (-0.12, 0.03)	0.22	4.19 (0.15)	-0.05 (-0.13, 0.03)	0.19
*APOE*3	4.19 (0.03)	Reference		4.24 (0.15)	Reference	
*APOE*4	4.19 (0.04)	0.002 (-0.05, 0.06)	0.93	4.20 (0.15)	-0.04 (-0.10, 0.02)	0.16
27HC						
*APOE*2	4.93 (0.04)	-0.05 (-0.12, 0.02)	0.18	4.96 (0.15)	-0.03 (-0.11, 0.05)	0.47
*APOE*3	4.98 (0.03)	Reference		4.99 (0.14)	Reference	
*APOE*4	5.01 (0.03)	0.03 (-0.02, 0.08)	0.28	4.99 (0.14)	0.003 (-0.05, 0.06)	0.92
24,25-EPOXY						
*APOE*2	27.22 (9.89)	12.62 (-4.03, 29.27)	0.14	34.90 (27.70)	**20.18 (1.54, 38.82)**	**0.03**
*APOE*3	14.60 (6.47)	Reference		14.70 (26.20)	Reference	
*APOE*4	7.05 (7.58)	-7.55 (-19.19, 4.09)	0.20	10.00 (26.00)	-4.70 (-18.06, 8.67)	0.49
24HC/27HC						
*APOE*2	0.84 (0.01)	-0.002 (-0.01, 0.01)	0.80	0.84 (0.03)	-0.01 (-0.02, 0.01)	0.34
*APOE*3	0.84 (0.005)	Reference		0.85 (0.02)	Reference	
*APOE*4	0.84 (0.01)	-0.004 (-0.01, 0.005)	0.34	0.84 (0.02)	-0.01 (-0.02, 0.001)	0.07
**Hormone**						
Estradiol						
*APOE*2	2.35 (0.06)	-0.005 (-0.10, 0.09)	0.93	2.30 (0.10)	-0.02 (-0.13, 0.09)	0.66
*APOE*3	2.36 (0.04)	Reference		2.32 (0.09)	Reference	
*APOE*4	2.43 (0.05)	0.07 (-0.0003, 0.14)	0.05	2.41 (0.10)	**0.09 (0.01, 0.17)**	**0.03**
Estradiol (B)						
*APOE*2	1.87 (0.06)	-0.05 (-0.15, 0.06)	0.40	1.85 (0.11)	-0.06 (-0.18, 0.05)	0.28
*APOE*3	1.91 (0.04)	Reference		1.91 (0.10)	Reference	
*APOE*4	1.96 (0.05)	0.05 (-0.03, 0.13)	0.19	1.99 (0.10)	0.08 (-0.01, 0.16)	0.07
Estrone						
*APOE*2	3.63 (0.16)	-0.09 (-0,32, 0.15)	0.46	3.49 (0.28)	-0.21 (-0.49, 0.06)	0.13
*APOE*3	3.71 (0.11)	Reference		3.70 (0.28)	Reference	
*APOE*4	3.66 (0.13)	-0.05 (-0.23, 0.13)	0.56	3.62 (0.29)	-0.09 (-0.30, 0.13)	0.43
Testosterone (F)						
*APOE*2	1.92 (0.20)	0.17 (-0.14, 0.48)	0.28	1.39 (0.33)	0.24 (-0.12, 0.60)	0.19
*APOE*3	1.75 (0.13)	Reference		1.15 (0.33)	Reference	
*APOE*4	1.78 (0.16)	0.03 (-0.20, 0.26)	0.81	1.22 (0.35)	0.08 (-0.19, 0.34)	0.57
Testosterone (B)						
*APOE*2	5.12 (0.20)	0.17 (-0.14, 0.48)	0.28	4.59 (0.33)	0.24 (-0.12, 0.60)	0.19

*APOE*3	4.95 (0.13)	Reference		4.35 (0.33)	Reference	
*APOE*4	4.98 (0.16)	0.03 (-0.20, 0.26)	0.81	4.43 (0.35)	0.08 (-0.19, 0.34)	0.57

Basic adjustment included age, body mass index, cholesterol-lowering medication, oophorectomy, hysterectomy, and hormone use. Multivariable adjustment further included education, smoking status, alcohol intake, hypertension, cardiovascular disease, stroke, and type 2 diabetes.
Abbreviations: 24,25-EPOXY, 24(S),25-epoxycholesterol; 24HC, 24(S)-hydroxycholesterol; 27HC, 27-hydroxycholesterol; *APOE*2, Apolipoprotein E ε2/ε2 or ε2/ε3 genotype; *APOE*3, Apolipoprotein E ε3/ε3 genotype; *APOE*4, Apolipoprotein E ε4/ε4 or ε4/ε3 genotype; B, bioavailable; CI, confidence interval; F, free; LSM, least-squares mean; SE, standard error.

Supplemental Table S6. Associations between cognitive status and oxysterols, hormones, reproductive characteristics, and *APOE*.

	3MS scores			
	**Basic adjustment**		**Multivariable adjustment**	
**Variable**	**β (95% CI)**	***p*-value**	**β (95% CI)**	***p*-value**
Oxysterol				
24HC	-1.32 (-2.98, 0.34)	0.12	-1.37 (-2.98, 0.24)	0.10
27HC	**-1.80 (-3.53, -0.07)**	**0.04**	-1.60 (-3.29, 0.10)	0.06
24,25-EPOXY	-0.001 (-0.02, 0.02)	0.88	-0.001 (-0.02, 0.02)	0.87
24HC/27HC	0.25 (-9.84, 10.35)	0.96	-1.34 (-11.34, 8.67)	0.79
Hormone				
Estradiol	-0.44 (-1.25, 0.36)	0.28	-0.22 (-1.00, 0.56)	0.58
Estradiol (B)	-0.36 (-1.11, 0.39)	0.35	-0.09 (-0.82, 0.65)	0.82
Estrone	2.11 (-0.71, 4.93)	0.14	2.64 (-0.56, 5.84)	0.10
Testosterone (F)	1.49 (-1.37, 4.35)	0.30	1.13 (-2.04, 4.31)	0.47
Testosterone (B)	1.49 (-1.37, 4.35)	0.30	1.13 (-2.04, 4.30)	0.47
Reproductive characteristic				
Age at menopause	0.04 (-0.01, 0.09)	0.10	0.01 (-0.04, 0.06)	0.58
Oophorectomy	-0.41 (-1.33, 0.52)	0.39	-0.51 (-1.43, 0.41)	0.28
Hormone use	1.44 (-0.03, 2.90)	0.05	1.22 (-0.25, 2.69)	0.10
Number of pregnancies	0.07 (-0.15, 0.29)	0.53	0.17 (-0.04, 0.38)	0.12
*APOE*4 status				
*APOE*4-	Reference		Reference	
*APOE*4+	**-0.96 (-1.74, -0.18)**	**0.02**	**-0.92 (-1.69, -0.15)**	**0.02**

Basic adjustment included age, body mass index, cholesterol-lowering medication, oophorectomy, hysterectomy, and hormone use. Multivariable adjustment further included education, smoking status, alcohol intake, hypertension, cardiovascular disease, stroke, and type 2 diabetes.
Abbreviations: 24,25-EPOXY, 24(S),25-epoxycholesterol; 24HC, 24(S)-hydroxycholesterol; 27HC, 27-hydroxycholesterol; 3MS = Modified Mini Mental State exam; *APOE*4+, Apolipoprotein E ε4 carrier; *APOE*4-, Apolipoprotein E ε4 non-carrier; B, bioavailable; CI, confidence interval; F, free.

Supplemental Table S7. Associations between sex hormones and oxysterols after further adjustment for waist circumference.

Hormone	24HC<br>β (95% CI)	*p*-value	27HC<br>β (95% CI)	*p*-value	24,25-EPOXY<br>β (95% CI)	*p*-value
Estradiol	0.02 (-0.02, 0.07)	0.26	0.02 (-0.02, 0.06)	0.31	-2.36 (-9.78, 5.05)	0.53
Estradiol * *APOE*4	0.003 (-0.10, 0.10)	0.95	0.08 (-0.01, 0.18)	0.09	15.12 (-10.06, 40.29)	0.24
Estradiol * cholesterol med.	-0.15 (-0.32, 0.03)	0.09	-0.10 (-0.27, 0.08)	0.27	-21.54 (-47.09, 4.01)	0.10
Estradiol (B)	0.04 (-0.001, 0.08)	0.06	**0.04 (0.003, 0.08)**	**0.04**	-2.59 (-9.80, 4.62)	0.48
Estradiol (B) * *APOE*4	0.004 (-0.01, 0.10)	0.93	0.08 (-0.01, 0.17)	0.08	12.60 (-10.18, 35.37)	0.28
Estradiol (B) * cholesterol med.	-0.12 (-0.29, 0.05)	0.17	-0.06 (-0.23, 0.11)	0.50	-19.62 (-44.42, 5.18)	0.12
Estrone	**0.30 (0.02, 0.59)**	**0.04**	0.22 (-0.03, 0.48)	0.09	19.95 (-47.07, 86.96)	0.55
Estrone * *APOE*4	-0.12 (-0.82, 0.58)	0.73	0.03 (-0.60, 0.66)	0.93	82.75 (-127.13, 292.63)	0.41
Estrone * cholesterol med.	0.09 (-1.11, 1.29)	0.88	0.23 (-0.83, 1.29)	0.67	8.49 (-234.96, 251.94)	0.94
Testosterone (F)	0.04 (-0.22, 0.30)	0.77	0.09 (-0.11, 0.30)	0.36	24.81 (-50.15, 99.77)	0.49
Testosterone (F) * *APOE*4	0.14 (-0.89, 1.18)	0.77	-0.02 (-0.87, 0.84)	0.97	-49.19 (-655.20, 556.82)	0.86
Testosterone (F) * cholesterol med.	-0.46 (-1.55, 0.62)	0.39	-0.74 (-1.55, 0.07)	0.07	-16.13 (-264.39, 232.14)	0.89
Testosterone (B)	0.04 (-0.22, 0.30)	0.76	0.09 (-0.11, 0.30)	0.35	24.79 (-50.17, 99.75)	0.49
Testosterone (B) * *APOE*4	0.14 (-0.89, 1.17)	0.78	-0.02 (-0.87, 0.84)	0.97	-49.53 (-656.04, 556.97)	0.86
Testosterone (B) * cholesterol med.	-0.46 (-1.55, 0.62)	0.39	-0.74 (-1.55, 0.07)	0.07	-16.10 (-264.58, 232.38)	0.89

Models were adjusted for age, body mass index, waist circumference, cholesterol-lowering medication, oophorectomy, hysterectomy, hormone use, education, smoking status, alcohol intake, hypertension, cardiovascular disease, stroke, and type 2 diabetes.
Abbreviations: 24,25-EPOXY, 24(S),25-epoxycholesterol; 24HC, 24(S)-hydroxycholesterol; 27HC, 27-hydroxycholesterol; *APOE*4, Apolipoprotein E ε4 carrier status; cholesterol med., cholesterol-lowering medication; B, bioavailable; CI, confidence interval; F, free.

Supplemental Table S8. Associations between sex hormones and oxysterols after further adjustment for cancer.

Hormone	24HC β (95% CI)	*p*-value	27HC β (95% CI)	*p*-value	24,25-EPOXY β (95% CI)	*p*-value
Estradiol	0.02 (-0.02, 0.07)	0.27	0.02 (-0.02, 0.07)	0.26	-2.31 (-9.73, 5.11)	0.54
Estradiol * *APOE*4	-0.001 (-0.10, 0.10)	0.99	0.09 (-0.002, 0.19)	0.06	15.33 (-9.45, 40.11)	0.22
Estradiol * cholesterol med.	-0.14 (-0.32, 0.03)	0.11	-0.09 (-0.26, 0.08)	0.31	-21.05 (-46.48, 4.39)	0.11
Estradiol (B)	**0.04 (0.001, 0.08)**	**0.046**	**0.05 (0.01, 0.09)**	**0.02**	-2.34 (-9.50, 4.83)	0.52
Estradiol (B) * *APOE*4	-0.001 (-0.09, 0.09)	0.98	**0.09 (0.005, 0.18)**	**0.04**	12.82 (-9.35, 34.99)	0.26
Estradiol (B) * cholesterol med.	-0.11 (-0.28, 0.06)	0.20	-0.05 (-0.22, 0.11)	0.53	-19.20 (-43.90, 5.50)	0.13
Estrone	**0.32 (0.03, 0.62)**	**0.03**	**0.27 (0.01, 0.52)**	**0.04**	14.48 (-54.20, 83.16)	0.67
Estrone * *APOE*4	-0.04 (-0.77, 0.68)	0.91	0.11 (-0.49, 0.72)	0.70	47.96 (-160.56, 256.47)	0.63
Estrone * cholesterol med.	0.12 (-1.08, 1.32)	0.84	0.17 (-0.86, 1.20)	0.74	17.04 (-225.18, 259.26)	0.89
Testosterone (F)	0.03 (-0.23, 0.29)	0.82	0.11 (-0.10, 0.31)	0.29	24.59 (-48.34, 97.52)	0.48
Testosterone (F) * *APOE*4	0.15 (-0.88, 1.18)	0.77	-0.04 (-0.89, 0.82)	0.93	-134.83 (-813.83, 544.17)	0.66
Testosterone (F) * cholesterol med.	-0.48 (1.56, 0.60)	0.37	-0.72 (-1.53, 0.10)	0.08	-20.65 (-268.12, 226.82)	0.86
Testosterone (B)	0.03 (-0.23, 0.29)	0.81	0.11 (-0.10, 0.31)	0.28	24.58 (-48.34, 97.49)	0.48
Testosterone (B) * *APOE*4	0.15 (-0.89, 1.18)	0.77	-0.04 (-0.89, 0.82)	0.93	134.97 (-814.30, 544.37)	0.66
Testosterone (B) * cholesterol med.	-0.48 (-1.56, 0.60)	0.37	-0.72 (-1.53, 0.10)	0.08	-20.63 (-268.33, 227.06)	0.86

Models were adjusted for age, body mass index, cholesterol-lowering medication, oophorectomy, hysterectomy, hormone use, education, smoking status, alcohol intake, hypertension, stroke, and type 2 diabetes.
Abbreviations: 24,25-EPOXY, 24(S),25-epoxycholesterol; 24HC, 24(S)-hydroxycholesterol; 27HC, 27-hydroxycholesterol; *APOE*4, Apolipoprotein E ε4 carrier status; cholesterol med., cholesterol-lowering medication; B, bioavailable; CI, confidence interval; F, free.

Supplemental Table S9. Associations between sex hormones and oxysterols according to *APOE*4 status and cholesterol medication use.

Hormone	24HC β (95% CI)	*p*-value	27HC β (95% CI)	*p*-value	24,25-EPOXY β (95% CI)	*p*-value
***APOE*4 carriers**						
Estradiol	0.10 (-0.01, 0.20)	0.07	**0.13 (0.02, 0.24)**	**0.02**	15.87 (-8.31, 40.06)	0.19
Estradiol (B)	**0.11 (0.01, 0.22)**	**0.04**	**0.16 (0.05, 0.27)**	**0.004**	14.07 (-8.86, 37.01)	0.22
Estrone	-0.37 (-2.58, 1.84)	0.54	1.01 (-0.39, 2.41)	0.10	NE	
Testosterone (F)	NE		NE		NE	
Testosterone (B)	NE		NE		NE	
***APOE*4 non-carriers**						
Estradiol	0.02 (-0.04, 0.09)	0.45	-0.01 (-0.07, 0.05)	0.75	-9.88 (-24.10, 4.33)	0.17
Estradiol (B)	0.03 (-0.03, 0.09)	0.36	0.003 (-0.05, 0.06)	0.91	-9.79 (-23.32, 3.74)	0.16
Estrone	0.30 (-0.16, 0.77)	0.19	0.33 (-0.002, 0.66)	0.05	-18.04 (-155.16, 119.07)	0.78
Testosterone (F)	0.07 (-0.34, 0.48)	0.73	0.16 (-0.13, 0.45)	0.26	21.25 (-122.16, 164.65)	0.74
Testosterone (B)	0.07 (-0.34, 0.48)	0.72	0.16 (-0.13, 0.46)	0.26	21.24 (-122.11, 164.59)	0.74
**Cholesterol medication users**						
Estradiol	-0.10 (-0.28, 0.08)	0.29	-0.06 (-0.27, 0.14)	0.53	**-24.01 (-47.47, -0.56)**	**0.045**
Estradiol (B)	-0.05 (-0.24, 0.14)	0.61	0.0001 (-0.22, 0.22)	1.00	**-27.54 (-52.05, -3.03)**	**0.03**
Estrone	NE		NE		NE	
Testosterone (F)	NE		NE		NE	
Testosterone (B)	NE		NE		NE	
**Cholesterol medication non-users**						
Estradiol	0.03 (-0.01, 0.08)	0.14	0.03 (-0.01, 0.08)	0.14	-0.43 (-8.24, 7.37)	0.91
Estradiol (B)	**0.05 (0.01, 0.09)**	**0.02**	**0.06 (0.01, 0.10)**	**0.008**	-0.59 (-8.11, 6.94)	0.88
Estrone	0.27 (-0.05, 0.59)	0.09	0.17 (-0.09, 0.43)	0.20	24.42 (-60.17, 109.01)	0.55
Testosterone (F)	0.04 (-0.23, 0.30)	0.79	0.12 (-0.06, 0.30)	0.18	27.10 (-53.82, 108.03)	0.48
Testosterone (B)	0.04 (-0.23, 0.30)	0.78	0.12 (-0.06, 0.30)	0.18	27.07 (-53.84, 107.99)	0.48

Models were adjusted for age, body mass index, cholesterol-lowering medication, oophorectomy, hysterectomy, hormone use, education, smoking status, alcohol intake, hypertension, cardiovascular disease, stroke, and type 2 diabetes.
Abbreviations: 24,25-EPOXY, 24(S),25-epoxycholesterol; 24HC, 24(S)-hydroxycholesterol; 27HC, 27-hydroxycholesterol; B, bioavailable; CI, confidence interval; F, free; NE, not estimable due to too few observations.

Supplemental Table S10. False Discovery Rate-adjusted *p*-values for associations between sex hormones and oxysterols.

Oxysterol	Hormone	Model type	β (95% CI)	Unadjusted *p*-value	FDR-adjusted *p*-value
24HC	Estradiol	Main model	0.02 (-0.02, 0.07)	0.27	0.59
		Interaction, *APOE*4	-0.001 (-0.10, 0.10)	0.98	0.98
		Stratified, *APOE*4+	0.10 (-0.01, 0.20)	0.07	0.38
		Stratified, *APOE*4-	0.02 (-0.04, 0.09)	0.45	0.90
		Interaction, cholesterol medication	-0.14 (-0.32, 0.03)	0.11	0.43
		Stratified, cholesterol medication users	-0.10 (-0.28, 0.08)	0.29	0.61
		Stratified, cholesterol medication non-users	0.03 (-0.01, 0.08)	0.14	0.43
	Estradiol (B)	Main model	**0.04 (0.001, 0.08)**	**0.046**	0.23
		Interaction, *APOE*4	-0.002 (-0.09, 0.09)	0.97	0.98
		Stratified, *APOE*4+	**0.11 (0.01, 0.22)**	**0.04**	0.36
		Stratified, *APOE*4-	0.03 (-0.03, 0.09)	0.36	0.76
		Interaction, cholesterol medication	-0.11 (-0.28, 0.06)	0.20	0.45
		Stratified, cholesterol medication users	-0.05 (-0.24, 0.14)	0.61	0.84
		Stratified, cholesterol medication non-users	**0.05 (0.01, 0.09)**	**0.02**	0.35
	Estrone	Main model	**0.31 (0.02, 0.59)**	**0.04**	0.23
		Interaction, *APOE*4	-0.06 (-0.77, 0.65)	0.87	0.98
		Stratified, *APOE*4+	-0.37 (-2.58, 1.84)	0.54	0.96
		Stratified, *APOE*4-	0.30 (-0.16, 0.77)	0.19	0.59
		Interaction, cholesterol medication	0.13 (-1.06, 1.32)	0.83	0.96
		Stratified, cholesterol medication users	NE		
		Stratified, cholesterol medication non-users	0.27 (-0.05, 0.59)	0.09	0.43
	Testosterone (F)	Main model	0.03 (-0.22, 0.29)	0.79	0.79
		Interaction, *APOE*4	0.15 (-0.86, 1.16)	0.77	0.96
		Stratified, *APOE*4+	NE		
		Stratified, *APOE*4-	0.07 (-0.34, 0.48)	0.73	0.96
		Interaction, cholesterol medication	-0.47 (-1.53, 0.60)	0.38	0.68
		Stratified, cholesterol medication users	NE		
		Stratified, cholesterol medication non-users	0.04 (-0.23, 0.30)	0.79	0.96
	Testosterone (B)	Main model	0.04 (-0.22, 0.29)	0.78	0.79
		Interaction, *APOE*4	0.15 (-0.87, 1.16)	0.77	0.96
		Stratified, *APOE*4+	NE		
		Stratified, *APOE*4-	0.07 (-0.34, 0.48)	0.72	0.96

		Interaction, cholesterol medication	-0.47 (-1.54, 0.60)	0.38	0.68
		Stratified, cholesterol medication users	NE		
		Stratified, cholesterol medication non-users	0.04 (-0.23, 0.30)	0.78	0.96
27HC	Estradiol	Main model	0.02 (-0.02, 0.07)	0.26	0.59
		Interaction, *APOE*4	0.09 (-0.003, 0.19)	0.06	0.36
		Stratified, *APOE*4+	**0.13 (0.02, 0.24)**	**0.02**	0.32
		Stratified, *APOE*4-	-0.01 (-0.07, 0.05)	0.75	0.96
		Interaction, cholesterol medication	-0.09 (-0.26, 0.08)	0.30	0.61
		Stratified, cholesterol medication users	-0.06 (-0.27, 0.14)	0.53	0.80
		Stratified, cholesterol medication non-users	0.03 (-0.01, 0.08)	0.14	0.43
	Estradiol (B)	Main model	**0.05 (0.01, 0.09)**	**0.02**	0.23
		Interaction, *APOE*4	**0.09 (0.004, 0.18)**	**0.04**	0.36
		Stratified, *APOE*4+	**0.16 (0.05, 0.27)**	**0.004**	0.15
		Stratified, *APOE*4-	0.003 (-0.05, 0.06)	0.91	0.98
		Interaction, cholesterol medication	-0.05 (-0.22, 0.11)	0.53	0.80
		Stratified, cholesterol medication users	0.0001 (-0.22, 0.22)	1.00	1.00
		Stratified, cholesterol medication non-users	**0.06 (0.01, 0.10)**	**0.008**	0.28
	Estrone	Main model	0.22 (-0.04, 0.47)	0.09	0.34
		Interaction, *APOE*4	0.02 (-0.60, 0.64)	0.94	0.98
		Stratified, *APOE*4+	1.01 (-0.39, 2.41)	0.10	0.50
		Stratified, *APOE*4-	0.33 (-0.002, 0.66)	0.05	0.36
		Interaction, cholesterol medication	0.19 (-0.86, 1.24)	0.72	0.96
		Stratified, cholesterol medication users	NE		
		Stratified, cholesterol medication non-users	0.17 (-0.09, 0.43)	0.20	0.45
	Testosterone (F)	Main model	0.10 (-0.10, 0.31)	0.32	0.59
		Interaction, *APOE*4	-0.03 (-0.89, 0.84)	0.95	0.98
		Stratified, *APOE*4+	NE		
		Stratified, *APOE*4-	0.16 (-0.13, 0.45)	0.26	0.59
		Interaction, cholesterol medication	-0.73 (-1.55, 0.08)	0.08	0.43
		Stratified, cholesterol medication users	NE		
		Stratified, cholesterol medication non-users	0.12 (-0.06, 0.30)	0.18	0.45
	Testosterone (B)	Main model	0.10 (-0.10, 0.31)	0.31	0.59
		Interaction, *APOE*4	-0.02 (-0.89, 0.84)	0.95	0.98
		Stratified, *APOE*4+	NE		
		Stratified, *APOE*4-	0.16 (-0.13, 0.46)	0.26	0.59

		Interaction, cholesterol medication	-0.74 (-1.55, 0.08)	0.08	0.43
		Stratified, cholesterol medication users	NE		
		Stratified, cholesterol medication non-users	0.12 (-0.06, 0.30)	0.18	0.45
24,25-EPOXY	Estradiol	Main model	-2.28 (-9.67, 5.10)	0.54	0.63
		Interaction, *APOE*4	15.31 (-9.42, 40.03)	0.22	0.59
		Stratified, *APOE*4+	15.87 (-8.31, 40.06)	0.19	0.59
		Stratified, *APOE*4-	-9.88 (-24.10, 4.33)	0.17	0.59
		Interaction, cholesterol medication	-21.06 (-46.46, 4.34)	0.10	0.43
		Stratified, cholesterol medication users	**-24.01 (-47.47, -0.56)**	**0.045**	0.41
		Stratified, cholesterol medication non-users	-0.43 (-8.24, 7.37)	0.91	0.97
	Estradiol (B)	Main model	-2.31 (-9.45, 4.82)	0.52	0.63
		Interaction, *APOE*4	12.76 (-9.35, 34.87)	0.26	0.59
		Stratified, *APOE*4+	14.07 (-8.86, 37.01)	0.22	0.59
		Stratified, *APOE*4-	-9.79 (-23.32, 3.74)	0.15	0.59
		Interaction, cholesterol medication	-19.21 (-43.88, 5.45)	0.13	0.43
		Stratified, cholesterol medication users	**-27.54 (-52.05, -3.03)**	**0.03**	0.35
		Stratified, cholesterol medication non-users	-0.59 (-8.11, 6.94)	0.88	0.96
	Estrone	Main model	20.60 (-44.74, 85.94)	0.52	0.63
		Interaction, *APOE*4	63.75 (-137.17, 264.66)	0.51	0.96
		Stratified, *APOE*4+	NE		
		Stratified, *APOE*4-	-18.04 (-155.16, 119.07)	0.78	0.96
		Interaction, cholesterol medication	5.38 (-231.09, 241.85)	0.96	0.99
		Stratified, cholesterol medication users	NE		
		Stratified, cholesterol medication non-users	24.42 (-60.17, 109.01)	0.55	0.80
	Testosterone (F)	Main model	25.69 (-44.38, 95.76)	0.45	0.63
		Interaction, *APOE*4	-71.93 (-623.94, 480.09)	0.78	0.96
		Stratified, *APOE*4+	NE		
		Stratified, *APOE*4-	21.25 (-122.16, 164.65)	0.74	0.96
		Interaction, cholesterol medication	-16.90 (-253.90, 220.11)	0.88	0.96
		Stratified, cholesterol medication users	NE		
		Stratified, cholesterol medication non-users	27.10 (-53.82, 108.03)	0.48	0.79
	Testosterone (B)	Main model	25.67 (-44.40, 95.73)	0.45	0.63
		Interaction, *APOE*4	-72.14 (-624.76, 480.48)	0.77	0.96
		Stratified, *APOE*4+	NE		
		Stratified, *APOE*4-	21.24 (-122.11, 164.59)	0.74	0.96

Interaction, cholesterol medication	-16.87 (-254.08, 220.35)	0.88	0.96
Stratified, cholesterol medication users	NE		
Stratified, cholesterol medication non-users	27.07 (-53.84, 107.99)	0.48	0.79

Models were adjusted for age, body mass index, cholesterol-lowering medication (except in interaction and stratified models by cholesterol-lowering medication use), oophorectomy, hysterectomy, hormone use, education, smoking status, alcohol intake, hypertension, cardiovascular disease, stroke, and type 2 diabetes.
Abbreviations: 24,25-EPOXY, 24(S),25-epoxycholesterol; 24HC, 24(S)-hydroxycholesterol; 27HC, 27-hydroxycholesterol; *APOE*4, Apolipoprotein E ε4 carrier status; B, bioavailable; cholesterol med., cholesterol-lowering medication; CI, confidence interval; F, free; NE, not estimable due to too few observations.

Supplemental Table S11. Associations between sex hormones and 24HC/27HC ratio.

Hormone	24HC/27HC<br>β (95% CI)	*p*-value
Estradiol	0.001 (-0.01, 0.01)	0.82
Estradiol * *APOE*4	**-0.02 (-0.03, -0.0002)**	**0.047**
Estradiol * cholesterol med.	-0.01 (-0.04, 0.01)	0.34
Estradiol (B)	0.0001 (-0.01, 0.01)	0.99
Estradiol (B) * *APOE*4	**-0.02 (-0.03, -0.001)**	**0.03**
Estradiol (B) * cholesterol med.	-0.01 (-0.04, 0.01)	0.31
Estrone	0.03 (-0.02, 0.08)	0.29
Estrone * *APOE*4	-0.01 (-0.12, 0.10)	0.83
Estrone * cholesterol med.	-0.01 (-0.21, 0.20)	0.95
Testosterone (F)	-0.01 (-0.05, 0.03)	0.63
Testosterone (F) * *APOE*4	0.04 (-0.13, 0.21)	0.63
Testosterone (F) * cholesterol med.	0.02 (-0.16, 0.20)	0.84
Testosterone (B)	-0.01 (-0.05, 0.03)	0.64
Testosterone (B) * *APOE*4	0.04 (-0.13, 0.21)	0.64
Testosterone (B) * cholesterol med.	0.02 (-0.16, 0.20)	0.85

Models were adjusted for age, body mass index, cholesterol-lowering medication, oophorectomy, hysterectomy, hormone use, education, smoking status, alcohol intake, hypertension, cardiovascular disease, stroke, and type 2 diabetes.
Abbreviations: 24HC, 24(S)-hydroxycholesterol; 27HC, 27-hydroxycholesterol; B, bioavailable; CI, confidence interval; F, free.

Supplemental Table S12. Associations between reproductive history and oxysterols after further adjustment for waist circumference.

	**24HC**		**27HC**		**24,25-EPOXY**	
**Reproductive history**	**β (95% CI)**	***p*-value**	**β (95% CI)**	***p*-value**	**β (95% CI)**	***p*-value**
Age at menopause	-0.0003 (-0.004, 0.003)	0.86	-0.002 (-0.005, 0.002)	0.38	-0.31 (-0.95, 0.32)	0.33
Age at menopause * *APOE*4	0.003 (-0.01, 0.01)	0.49	0.001 (-0.01, 0.01)	0.88	-1.81 (-3.78, 0.16)	0.07
Age at menopause * cholesterol med.	**-0.01 (-0.02, -0.0001)**	**0.03**	-0.004 (-0.01, 0.01)	0.41	-0.02 (-1.59, 1.55)	0.98
Oophorectomy	-0.01 (-0.07, 0.05)	0.67	0.01 (-0.04, 0.07)	0.69	7.84 (-2.46, 18.13)	0.14
Oophorectomy * *APOE*4	0.02 (-0.09, 0.14)	0.68	-0.07 (-0.18, 0.04)	0.21	-3.58 (-31.53, 24.37)	0.80
Oophorectomy * cholesterol med.	0.12 (-0.04, 0.28)	0.14	0.07 (-0.09, 0.23)	0.38	3.51 (-24.32, 31.34)	0.80
Current hormone use	0.002 (-0.08, 0.08)	0.97	-0.03 (-0.11, 0.04)	0.42	-11.49 (-26.36, 3.39)	0.13
Current hormone use * *APOE*4	0.001 (-0.20, 0.21)	0.99	-0.08 (-0.27, 0.12)	0.43	13.63 (-37.69, 64.94)	0.60
Current hormone use * cholesterol med.	0.51 (-0.05, 1.08)	0.07	0.36 (-0.19, 0.90)	0.20	13.77 (-66.16, 93.70)	0.74
Number of pregnancies	0.001 (-0.01, 0.02)	0.86	-0.003 (-0.02, 0.01)	0.70	0.72 (-1.87, 3.31)	0.59
Number of pregnancies * *APOE*4	-0.01 (-0.04, 0.03)	0.71	0.01 (-0.02, 0.05)	0.56	3.27 (-5.49, 12.03)	0.46
Number of pregnancies * cholesterol med.	0.03 (-0.03, 0.08)	0.34	0.01 (-0.04, 0.06)	0.57	2.38 (-6.41, 11.17)	0.60

Models were adjusted for age, body mass index, waist circumference, cholesterol-lowering medication, oophorectomy, hysterectomy, hormone use, education, smoking status, alcohol intake, hypertension, cardiovascular disease, stroke, and type 2 diabetes.
Abbreviations: 24,25-EPOXY, 24(S),25-epoxycholesterol; 24HC, 24(S)-hydroxycholesterol; 27HC, 27-hydroxycholesterol; *APOE*4, Apolipoprotein E ε4 carrier status; B, bioavailable; cholesterol med., cholesterol-lowering medication; CI, confidence interval; F, free.

Supplemental Table S13. Associations between reproductive history and oxysterols after further adjustment for cancer.

Reproductive history	24HC β (95% CI)	*p*-value	27HC β (95% CI)	*p*-value	24,25-EPOXY β (95% CI)	*p*-value
Age at menopause	-0.0003 (-0.004, 0.003)	0.85	-0.002 (-0.01, 0.001)	0.24	-0.34 (-0.96, 0.29)	0.29
Age at menopause * *APOE*4	0.004 (-0.01, 0.01)	0.42	-0.001 (-0.01, 0.01)	0.88	-1.73 (-3.67, 0.20)	0.08
Age at menopause * cholesterol med.	**-0.01 (-0.02, -0.001)**	**0.03**	-0.004 (-0.01, 0.01)	0.42	-0.03 (-1.60, 1.54)	0.97
Oophorectomy	-0.01 (-0.07, 0.04)	0.64	0.01 (-0.05, 0.07)	0.69	7.94 (-2.36, 18.23)	0.13
Oophorectomy * *APOE*4	0.02 (-0.10, 0.14)	0.75	-0.07 (-0.18, 0.04)	0.23	-3.87 (-31.51, 23.78)	0.78
Oophorectomy * cholesterol med.	0.13 (-0.04, 0.29)	0.13	0.08 (-0.08, 0.23)	0.35	3.73 (-23.98, 31.43)	0.79
Current hormone use	0.002 (-0.08, 0.08)	0.95	-0.03 (-0.10, 0.05)	0.47	-11.28 (-26.13, 3.56)	0.14
Current hormone use * *APOE*4	0.005 (-0.20, 0.21)	0.97	-0.07 (-0.26, 0.13)	0.50	13.71 (-37.60, 65.03)	0.60
Current hormone use * cholesterol med.	0.52 (-0.05, 1.08)	0.07	0.36 (-0.19, 0.91)	0.20	13.82 (-65.94, 93.58)	0.73
Number of pregnancies	0.002 (-0.01, 0.02)	0.76	-0.002 (-0.02, 0.01)	0.77	0.66 (-1.92, 3.24)	0.62
Number of pregnancies * *APOE*4	-0.004 (-0.04, 0.03)	0.85	0.01 (-0.03, 0.04)	0.63	3.26 (-5.50, 12.02)	0.47
Number of pregnancies * cholesterol med.	0.02 (-0.03, 0.08)	0.36	0.01 (-0.04, 0.06)	0.60	2.25 (-6.50, 11.00)	0.61

Models were adjusted for age, body mass index, cholesterol-lowering medication, oophorectomy, hysterectomy, hormone use, education, smoking status, alcohol intake, hypertension, stroke, and type 2 diabetes.
Abbreviations: 24,25-EPOXY, 24(S),25-epoxycholesterol; 24HC, 24(S)-hydroxycholesterol; 27HC, 27-hydroxycholesterol; *APOE*4, Apolipoprotein E ε4 carrier status; B, bioavailable; cholesterol med., cholesterol-lowering medication; CI, confidence interval; F, free.

Supplemental Table S14. Associations between reproductive history and oxysterols according to *APOE*4 status and cholesterol medication use.

Reproductive history	**24HC** β (95% CI)	*p*-value	**27HC** β (95% CI)	*p*-value	**24,25-EPOXY** β (95% CI)	*p*-value
***APOE*4 carriers**						
Age at menopause	0.004 (-0.004, 0.01)	0.29	-0.004 (-0.01, 0.005)	0.41	**-2.16 (-3.48, -0.85)**	**0.002**
Oophorectomy	-0.04 (-0.16, 0.09)	0.56	-0.06 (-0.19, 0.07)	0.37	4.51 (-22.79, 31.80)	0.74
Current hormone use	-0.003 (-0.18, 0.18)	0.98	-0.07 (-0.26, 0.12)	0.47	-9.19 (-50.87, 32.49)	0.66
Number of pregnancies	0.002 (-0.03, 0.04)	0.91	0.01 (-0.03, 0.04)	0.67	1.22 (-6.02, 8.47)	0.74
***APOE*4 non-carriers**						
Age at menopause	-0.001 (-0.01, 0.004)	0.64	-0.002 (-0.01, 0.002)	0.35	0.01 (-1.17, 1.20)	0.98
Oophorectomy	0.01 (-0.08, 0.09)	0.90	0.04 (-0.04, 0.11)	0.35	11.70 (-6.75, 30.15)	0.21
Current hormone use	0.04 (-0.07, 0.15)	0.46	0.03 (-0.07, 0.13)	0.50	-17.34 (-45.79, 11.11)	0.23
Number of pregnancies	0.003 (-0.02, 0.02)	0.78	-0.003 (-0.02, 0.01)	0.77	0.24 (-4.31, 4.80)	0.92
**Cholesterol medication users**						
Age at menopause	**-0.01 (-0.02, -0.002)**	**0.02**	-0.01 (-0.02, 0.005)	0.21	-0.44 (-2.22, 1.33)	0.61
Oophorectomy	0.21 (-0.04, 0.46)	0.10	0.17 (-0.11, 0.46)	0.23	20.63 (-15.32, 56.58)	0.25
Current hormone use	0.46 (-0.13, 1.06)	0.12	0.16 (-0.52, 0.83)	0.64	13.38 (-62.44, 89.19)	0.72
Number of pregnancies	0.04 (-0.02, 0.10)	0.17	0.03 (-0.04, 0.09)	0.43	1.99 (-7.03, 11.00)	0.66
**Cholesterol medication non-users**						
Age at menopause	0.001 (-0.003, 0.005)	0.58	-0.002 (-0.01, 0.002)	0.40	-0.35 (-1.04, 0.33)	0.31
Oophorectomy	-0.03 (-0.09, 0.03)	0.38	0.002 (-0.06, 0.06)	0.95	7.35 (-3.59, 18.29)	0.19
Current hormone use	-0.01 (-0.09, 0.07)	0.88	-0.03 (-0.11, 0.04)	0.37	-12.32 (-27.63, 2.99)	0.11
Number of pregnancies	0.0004 (-0.01, 0.02)	0.96	-0.003 (-0.02, 0.01)	0.69	0.42 (-2.32, 3.16)	0.76

Models were adjusted for age, body mass index, cholesterol-lowering medication, oophorectomy, hysterectomy, hormone use, education, smoking status, alcohol intake, hypertension, cardiovascular disease, stroke, and type 2 diabetes.
Abbreviations: 24,25-EPOXY, 24(S),25-epoxycholesterol; 24HC, 24(S)-hydroxycholesterol; 27HC, 27-hydroxycholesterol; CI, confidence interval.

Supplemental Table S15. False Discovery Rate-adjusted *p*-values for associations between reproductive variables and oxysterols.

Oxysterol	Reproductive characteristic	Model type	β (95% CI)	Unadjusted *p*-value	FDR-adjusted *p*-value
24HC	Age at menopause	Main model	-0.0003 (-0.004, 0.003)	0.86	0.93
		Interaction, *APOE*4	0.004 (-0.005, 0.01)	0.41	0.99
		Stratified, *APOE*4+	0.004 (-0.004, 0.01)	0.29	0.99
		Stratified, *APOE*4-	-0.001 (-0.01, 0.004)	0.64	0.99
		Interaction, cholesterol medication	**-0.01 (-0.02, -0.001)**	**0.03**	0.48
		Stratified, cholesterol medication users	**-0.01 (-0.02, -0.002)**	**0.02**	0.48
		Stratified, cholesterol medication non-users	0.001 (-0.003, 0.005)	0.58	0.87
	Oophorectomy	Main model	-0.01 (-0.07, 0.04)	0.64	0.93
		Interaction, *APOE*4	0.02 (-0.10, 0.14)	0.74	0.99
		Stratified, *APOE*4+	-0.04 (-0.16, 0.09)	0.56	0.99
		Stratified, *APOE*4-	0.01 (-0.08, 0.09)	0.90	0.99
		Interaction, cholesterol medication	0.13 (-0.04, 0.29)	0.13	0.66
		Stratified, cholesterol medication users	0.21 (-0.04, 0.46)	0.10	0.66
		Stratified, cholesterol medication non-users	-0.03 (-0.09, 0.03)	0.38	0.74
	Current hormone use	Main model	0.003 (-0.08, 0.08)	0.95	0.95
		Interaction, *APOE*4	0.002 (-0.20, 0.21)	0.99	0.99
		Stratified, *APOE*4+	-0.003 (-0.18, 0.18)	0.98	0.99
		Stratified, *APOE*4-	0.04 (-0.07, 0.15)	0.46	0.99
		Interaction, cholesterol medication	0.52 (-0.05, 1.08)	0.07	0.66
		Stratified, cholesterol medication users	0.46 (-0.13, 1.06)	0.12	0.66
		Stratified, cholesterol medication non-users	-0.01 (-0.09, 0.07)	0.88	0.96
	Number of pregnancies	Main model	0.002 (-0.01, 0.02)	0.75	0.93
		Interaction, *APOE*4	-0.004 (-0.04, 0.03)	0.82	0.99
		Stratified, *APOE*4+	0.002 (-0.03, 0.04)	0.91	0.99
		Stratified, *APOE*4-	0.003 (-0.02, 0.02)	0.78	0.99
		Interaction, cholesterol medication	0.02 (-0.03, 0.08)	0.37	0.74
		Stratified, cholesterol medication users	0.04 (-0.02, 0.10)	0.17	0.68
		Stratified, cholesterol medication non-users	0.0004 (-0.01, 0.02)	0.96	0.98
27HC	Age at menopause	Main model	-0.002 (-0.01, 0.001)	0.24	0.91
		Interaction, *APOE*4	-0.001 (-0.01, 0.01)	0.89	0.99
		Stratified, *APOE*4+	-0.004 (-0.01, 0.005)	0.41	0.99

		Stratified, *APOE*4-	-0.002 (-0.01, 0.002)	0.35	0.99
		Interaction, cholesterol medication	-0.004 (-0.01, 0.01)	0.42	0.74
		Stratified, cholesterol medication users	-0.01 (-0.02, 0.005)	0.21	0.68
		Stratified, cholesterol medication non-users	-0.002 (-0.01, 0.002)	0.40	0.74
	Oophorectomy	Main model	0.01 (-0.04, 0.07)	0.69	0.93
		Interaction, *APOE*4	-0.07 (-0.18, 0.04)	0.24	0.99
		Stratified, *APOE*4+	-0.06 (-0.19, 0.07)	0.37	0.99
		Stratified, *APOE*4-	0.04 (-0.04, 0.11)	0.35	0.99
		Interaction, cholesterol medication	0.08 (-0.08, 0.23)	0.35	0.74
		Stratified, cholesterol medication users	0.17 (-0.11, 0.46)	0.23	0.69
		Stratified, cholesterol medication non-users	0.002 (-0.06, 0.06)	0.95	0.98
	Current hormone use	Main model	-0.03 (-0.10, 0.05)	0.47	0.93
		Interaction, *APOE*4	-0.07 (-0.27, 0.13)	0.48	0.99
		Stratified, *APOE*4+	-0.07 (-0.26, 0.12)	0.47	0.99
		Stratified, *APOE*4-	0.03 (-0.07, 0.13)	0.50	0.99
		Interaction, cholesterol medication	0.36 (-0.19, 0.91)	0.20	0.68
		Stratified, cholesterol medication users	0.16 (-0.52, 0.83)	0.64	0.87
		Stratified, cholesterol medication non-users	-0.03 (-0.11, 0.04)	0.37	0.74
	Number of pregnancies	Main model	-0.002 (-0.02, 0.01)	0.78	0.93
		Interaction, *APOE*4	0.01 (-0.03, 0.04)	0.66	0.99
		Stratified, *APOE*4+	0.01 (-0.03, 0.04)	0.67	0.99
		Stratified, *APOE*4-	-0.003 (-0.02, 0.01)	0.77	0.99
		Interaction, cholesterol medication	0.01 (-0.04, 0.06)	0.60	0.87
		Stratified, cholesterol medication users	0.03 (-0.04, 0.09)	0.43	0.74
		Stratified, cholesterol medication non-users	-0.003 (-0.02, 0.01)	0.69	0.88
24,25-EPOXY	Age at menopause	Main model	-0.33 (-0.95, 0.30)	0.30	0.91
		Interaction, *APOE*4	-1.73 (-3.65, 0.19)	0.08	0.99
		Stratified, *APOE*4+	**-2.16 (-3.48, -0.85)**	**0.002**	0.06
		Stratified, *APOE*4-	0.01 (-1.17, 1.20)	0.98	0.99
		Interaction, cholesterol medication	-0.02 (-1.58, 1.54)	0.98	0.98
		Stratified, cholesterol medication users	-0.44 (-2.22, 1.33)	0.61	0.87
		Stratified, cholesterol medication non-users	-0.35 (-1.04, 0.33)	0.31	0.74
	Oophorectomy	Main model	7.85 (-2.42, 18.11)	0.13	0.80
		Interaction, *APOE*4	-3.90 (-31.49, 23.68)	0.78	0.99
		Stratified, *APOE*4+	4.51 (-22.79, 31.80)	0.74	0.99

	Stratified, *APOE*4-	11.70 (-6.75, 30.15)	0.21	0.99
	Interaction, cholesterol medication	3.85 (-23.81, 31.51)	0.79	0.88
	Stratified, cholesterol medication users	20.63 (-15.32, 56.58)	0.25	0.69
	Stratified, cholesterol medication non-users	7.35 (-3.59, 18.29)	0.19	0.68
Current hormone use	Main model	-11.35 (-26.17, 3.47)	0.13	0.80
	Interaction, *APOE*4	13.83 (-37.29, 64.94)	0.59	0.99
	Stratified, *APOE*4+	-9.19 (-50.87, 32.49)	0.66	0.99
	Stratified, *APOE*4-	-17.34 (-45.79, 11.11)	0.23	0.99
	Interaction, cholesterol medication	13.82 (-65.85, 93.50)	0.73	0.88
	Stratified, cholesterol medication users	13.38 (-62.44, 89.19)	0.72	0.88
	Stratified, cholesterol medication non-users	-12.32 (-27.63, 2.99)	0.11	0.66
Number of pregnancies	Main model	0.69 (-1.88, 3.25)	0.60	0.93
	Interaction, *APOE*4	3.23 (-5.43, 11.88)	0.46	0.99
	Stratified, *APOE*4+	1.22 (-6.02, 8.47)	0.74	0.99
	Stratified, *APOE*4-	0.24 (-4.31, 4.80)	0.92	0.99
	Interaction, cholesterol medication	2.24 (-6.50, 10.98)	0.61	0.87
	Stratified, cholesterol medication users	1.99 (-7.03, 11.00)	0.66	0.87
	Stratified, cholesterol medication non-users	0.42 (-2.32, 3.16)	0.76	0.88

Models were adjusted for age, body mass index, cholesterol-lowering medication (except in interaction and stratified models by cholesterol-lowering medication use), oophorectomy, hysterectomy, hormone use, education, smoking status, alcohol intake, hypertension, cardiovascular disease, stroke, and type 2 diabetes.
Abbreviations: 24,25-EPOXY, 24(S),25-epoxycholesterol; 24HC, 24(S)-hydroxycholesterol; 27HC, 27-hydroxycholesterol; *APOE*4, Apolipoprotein E ε4 carrier status; CI, confidence interval.

Supplemental Table S16. Associations between reproductive history and 24HC/27HC ratio.

Reproductive history	24HC/27HC β (95% CI)	*p*-value
Age at menopause	0.0003 (-0.0003, 0.001)	0.34
Age at menopause * *APOE*4	0.001 (-0.001, 0.002)	0.23
Age at menopause * cholesterol med.	-0.002 (-0.003, 0.0001)	0.06
Oophorectomy	-0.004 (-0.01, 0.005)	0.35
Oophorectomy * *APOE*4	0.02 (-0.003, 0.03)	0.11
Oophorectomy * cholesterol med.	0.01 (-0.01, 0.04)	0.35
Current hormone use	0.01 (-0.01, 0.02)	0.44
Current hormone use * *APOE*4	0.01 (-0.02, 0.04)	0.49
Current hormone use * cholesterol med.	0.04 (-0.05, 0.13)	0.39
Number of pregnancies	0.001 (-0.002, 0.003)	0.53
Number of pregnancies * *APOE*4	-0.002 (-0.01, 0.004)	0.47
Number of pregnancies * cholesterol med.	0.003 (-0.01, 0.01)	0.54

Models were adjusted for age, body mass index, cholesterol-lowering medication, oophorectomy, hysterectomy, hormone use, education, smoking status, alcohol intake, hypertension, cardiovascular disease, stroke, and type 2 diabetes.
Abbreviations: 24,25-EPOXY, 24(S),25-epoxycholesterol; 24HC, 24(S)-hydroxycholesterol; 27HC, 27-hydroxycholesterol; CI, confidence interval.
